# Exploration of international differences in preoperative characteristics, indications, postoperative management and endoscopic recurrence rates in patients with Crohn’s disease following ileocolic (re-)resection: first outcomes from the IMPACT consortium

**DOI:** 10.1093/ecco-jcc/jjag137

**Published:** 2026-10-23

**Authors:** Michiel T J Bak, Nassim Hammoudi, Sun-Ho Lee, Léon Maggiori, Oddeke van Ruler, Madeleine Bezault, Cristian Hernandez-Rocha, Annemarie C de Vries, Mark S Silverberg, Matthieu Allez, IMPACT (International Multidisciplinary Research Collaboration on Postoperative IBD) consortium; on behalf of the Dutch Initiative on Crohn and Colitis and the RAP-CD study group; on behalf of the NIDDK Inflammatory Bowel Disease Genetics Consortium; on behalf of the REMIND study group, Mariëlle Romberg-Camps, Evelien M J Beelen, Sander van der Marel, Frank Hoentjen, Rachel West, Marjolijn Duijvestein, P W Jeroen Maljaars, Jarmila D W van der Bilt, Christianne J Buskens, Laurents P S Stassen, Sita Jansen, Nanne K H de Boer, Carmen S Horjus, Laurents P S Stassen, Fiona D M van Schaik, Bindia J H Jharap, Gerard Dijkstra, John Rioux, Dermot McGovern, Richard Duerr, Judy Cho, Steven Brant, Mark Lazarev, Philippe Seksik, Stéphane Nancey, Anthony Buisson, Franck Carbonnel, Laurent Peyrin-Biroulet, Edouard Louis, Xavier Hébuterne, Mélanie Serrero, Arnaud Bourreille, Xavier Treton, Pauline Wils, Mathurin Fumery, Pierre Blanc, Mathieu Uzzan, Nicolas Barnich, Madeleine Bezault

**Affiliations:** Department of Gastroenterology and Hepatology, Erasmus University Medical Center, Rotterdam, the Netherlands; Zane Cohen Centre for Digestive Diseases, Mount Sinai Hospital, Toronto, Canada; Lunenfeld-Tanenbaum Research Institute, Mount Sinai Hospital, Toronto, Canada; Department of Gastroenterology, Hôpital Saint-Louis, Paris, France; Zane Cohen Centre for Digestive Diseases, Mount Sinai Hospital, Toronto, Canada; Lunenfeld-Tanenbaum Research Institute, Mount Sinai Hospital, Toronto, Canada; Surgery Department, Hôpital Saint-Louis, Paris, France; Department of Gastroenterology and Hepatology, Erasmus University Medical Center, Rotterdam, the Netherlands; Department of Surgery, IJsselland Hospital, Capelle aan den IJssel, the Netherlands; Department of Gastroenterology, Hôpital Saint-Louis, Paris, France; Zane Cohen Centre for Digestive Diseases, Mount Sinai Hospital, Toronto, Canada; Lunenfeld-Tanenbaum Research Institute, Mount Sinai Hospital, Toronto, Canada; Department of Gastroenterology, School of Medicine, Pontificia Universidad Católica de Chile, Santiago, Chile; Department of Gastroenterology and Hepatology, Erasmus University Medical Center, Rotterdam, the Netherlands; Zane Cohen Centre for Digestive Diseases, Mount Sinai Hospital, Toronto, Canada; Lunenfeld-Tanenbaum Research Institute, Mount Sinai Hospital, Toronto, Canada; Department of Gastroenterology, Hôpital Saint-Louis, Paris, France; Division of Gastroenterology and Hepatology, McGill University Health Center, Montreal, ON, Canada

**Keywords:** Crohn's disease, surgery, endoscopic postoperative recurrence

## Abstract

**Background:**

Reports on postoperative outcomes after ileocolic resection (ICR) in Crohn’s disease (CD) vary widely. To provide insights into these differences, we compared international data on preoperative characteristics and postoperative management and outcome.

**Methods:**

CD patients undergoing ICR were included from prospective cohort studies in the Netherlands (NL), France (FR), Canada (CAN), and United States of America (USA).

**Results:**

Among 1761 patients, 79% underwent primary resection. In this population, surgery for Montreal classification B1 disease was more common (22% vs 0%-5%) and performed after shorter disease duration (4.3 vs 6.2-7.1 years) in NL than in other cohorts. Active smoking was more prevalent in NL and FR than in CAN and USA (23%-34% vs 7%-13%). In CAN, 54% were biologic-naive versus 27%-36% in other cohorts. Postoperative prophylaxis was prescribed in 28% of patients in NL and CAN, compared with 48% in FR and USA. Ileocolonoscopy within 12 months was performed in 72% in USA versus 88% in other cohorts. In the re-resection population, B1 disease at surgery and disease duration did not differ, whereas other findings were similar. Endoscopic postoperative recurrence (modified Rutgeerts’ score ≥ i2b) varied from 36% in FR to 41% in NL, 15% in USA to 24% in CAN after primary resection and from 43% in NL, 46% in FR, 26% in CAN to 29% in USA cohort after re-resection.

**Conclusions:**

Notable differences in preoperative characteristics and postoperative management and outcomes for ileocolic (re-)resections in CD were observed across European and North American countries.

## 1. Introduction

Intestinal surgery is an important treatment modality for patients with Crohn’s disease (CD) and performed in approximately 25% of patients within 10 years after diagnosis.^1^ Ileocolic resection (ICR) is the most common type of intestinal resection in this population due to the frequent involvement of the terminal ileum, with or without cecal involvement. Surgery in CD is often reserved for treatment-refractory disease and disease complications, such as intra-abdominal abscesses and strictures, but it is also a viable treatment option for non-complicated terminal ileitis.^2^ Although intestinal surgery can induce disease remission and improve quality of life, it is not curative in the setting of CD.^3^ Postoperative recurrence (POR) is common and has been reported in approximately 1 out of 3 patients at 6 months postoperatively.^4-7^

To prevent POR, the current European guidelines recommend initiating postoperative prophylactic medication in patients considered to be “high-risk” whereas the American guidelines suggest treating all patients postoperatively, with the option to withhold prophylactic medication in those deemed to be “low-risk.”^8-10^ Aside from active smoking, no risk factors for POR have been consistently identified.^11^ In addition, male sex and a history of intestinal resection have been reported as risk factors in contemporary literature.^12^ As a result, risk stratification in the postoperative CD setting remains challenging and the question of which patients require postoperative prophylactic therapy is still unanswered.

In currently available literature, postoperative outcomes vary widely, for instance, the risk of endoscopic POR (ePOR) which is a widely accepted outcome measure after ICR in CD. Understanding these variations are critical. However, robust international comparative data on preoperative characteristics and postoperative management remain limited. These data are essential for assessing whether observed differences in outcomes are attributable to patient factors, disease characteristics or differences in medical and surgical management. In this study, we aimed to gain further insights in perioperative characteristics, postoperative management and ePOR outcomes following ileocolic (re-)resection (ICR) among patient cohorts from the Netherlands (NL), France (FR), Canada (CAN), and the United States of America (USA). In addition, we sought to explore whether the ePOR risk differed between countries after adjusting for potential confounding factors.

## 2. Methods

### 2.1 Study design and population

This study was performed within the IMPACT consortium (International Multidisciplinary Research Collaboration on Postoperative IBD), an intercontinental initiative focused on postoperative inflammatory bowel disease research that involves gastroenterologists, surgeons, and translational scientists. Patients with confirmed ileal CD who underwent ICR between December 2009 and May 2025 were identified from 3 ongoing prospective cohort studies in the NL, FR, and North America (CAN and the USA) with comparable datasets. Inclusion and exclusion criteria of the separate cohorts are described in Supplementary File 1.^4-6^

### 2.2 Data collection and endoscopic assessment

Data were prospectively extracted from the individual electronic patient files including demographics (sex, year of birth, smoking behavior), CD-related data (use and type of preoperative CD medication, prior intestinal resection, Montreal ­classification), type of postoperative prophylactic medication (immunomodulators or biologicals; including date of initiation) and performance of a postoperative ileocolonoscopy. Endoscopic severity was scored using the modified Rutgeerts’ score (mRS). In the Dutch and French cohort, the mRS was assessed by at least one least blinded physician based on the available endoscopic videos, images or reports, while in the North American cohort the mRS was assessed by a blinded physician using the endoscopic reports. Prior to merging the separate databases into a single dataset, several consensus meetings were organized to align definitions and variable coding to ensure consistency and comparability across the cohorts.

### 2.3 Outcomes

The study outcomes were the presence of ePOR, defined as a mRS of ≥ i2b, and severe ePOR, defined as mRS ≥ i3, at the first postoperative ileocolonoscopy performed within 1 year after surgery. Additionally, we aimed to compare perioperative characteristics, risk profiles for POR (as defined by European guidelines), initiation of postoperative prophylactic therapy (within 12 weeks of ICR), performance of postoperative ileocolonoscopy within 1 year (regardless of the mRS), and rates of (severe) ePOR between the involved countries, specifically between patients who underwent a primary resection or a re-resection.^9^ Patients with a primary resection or a re-resection were assessed in separate analyses. Only data available across all cohorts were presented and compared.

### 2.4 Statistical analysis

Descriptive statistics were used to describe the study sample, with categorical variables expressed as absolute numbers and percentages, and continuous variables as median with the first and third quartile (Q1 and Q3). Pairwise comparisons between the involved countries were performed using the chi-square test for categorical variables and the Mann-Whitney U test for continuous variables. No corrections for multiple testing were made.

Multivariable logistic regression was performed to identify whether the risk for ePOR and severe ePOR at first postoperative ileocolonoscopy differed per country, regardless whether they underwent primary resection or a re-resection. Based on the current literature and consensus among the study group and after assessment of potential multicollinearity, these models included male sex, age at surgery, disease duration, prior small bowel resection, preoperative lines of biologicals, disease location and behavior at surgery (Montreal L/B/p+), initiation of postoperative prophylactic medication, the time to first postoperative ileocolonoscopy, and year of surgery.

We performed a complete-case analysis to handle missing data in demographic and clinical parameters resulting in an analysis with 95.9% (1286/1341) of the eligible patients (ie, patients who underwent ileocolonoscopy <1 year post-surgery and who had an available mRS). Results are presented in adjusted odds ratios (aOR) and the corresponding 95% confidence intervals (CIs). Data was prepared in using SPSS (version 21, IBM, Chicago, IL) and analyzed in R software version 4.2.2 using the R package *geepack*.^13^

### 2.5 Ethics

The study was conducted in accordance with the Declaration of Helsinki. Informed consent was obtained from every participant prior to the inclusion of the study. The study was approved by the institutional research ethics board approval in every participating institution.

## 3. Results

### 3.1 Patient demographics and disease characteristics at surgery—primary resections

A total of 1761 patients with CD who underwent ICR were included (496 NL [28%], 688 FR [46%], 148 CAN [8%], USA 308 [18%]). 1389 patients (79%) underwent a primary resection (Table 1; comparative outcomes are presented in Tables S1a-1f). The rate of male sex was significantly lower in the Dutch cohort as compared to the other cohorts (40% vs FR 49%, CAN 55%, USA 52%, *P* ≤ .016). The median age at surgery in the Dutch cohort was higher (32 years) as compared to the Canadian (32 years) and American cohort (30 years; *P* ≤ .044). Other significant differences comprised a shorter disease duration in the Dutch cohort (4 years vs 6-7 years; *P* ≤ .001), higher (former or active) smoking rates (46%-58% vs 20%-25%; *P* ≤ .002) in the European countries, higher preoperative biological exposure (64%-73% vs 46%, *P* ≤ .011) in the European countries and the USA as compared to CAN, whereas a significantly higher rate of preoperative exposure to ustekinumab was observed in the Dutch cohort (20% vs 6%-13%; *P* ≤ .050) and a significant lower rate of anti-TNF agents in CAN (43% vs 63%-72%; *P* ≤ .007). Furthermore, significantly higher rates of ileocolic disease (Montreal L3) were reported in the American cohort as compared to the European cohorts (46% vs 33%-35%; *P* ≤ .003) and a significant difference was observed in disease behavior (higher rate of B1 disease and a lower rate of B3 disease) in the Dutch cohort as compared to the other cohorts (*P* < .001).

**Table 1.** Baseline characteristics of patients who underwent primary resection in the study cohort and per country.

Variables	Study cohort (*n* = 1389)	Dutch cohort (*n* = 401)	French cohort (*n* = 663)	Canadian cohort (*n* = 103)	American cohort (*n* = 222)	*P*-value*
**Male sex, *n (%)***	607 (46.9)	161 (40.1)	326 (49.2)	57 (55.3)	115 (52.3)	.008^a–c^
**Age at surgery (years), median (IQR)**	31.9 (24.9 - 43.8)	32.3 (24.8 - 48.0)	32.1 (25.6 - 42.9)	31.9 (23.6 - 38.2)	29.8 (24.6 - 41.7)	.044^b^ ^,^ ^c^
**Smoking status at surgery, *n (%)***						<.001^a–e^
***Never***	693 (54.5)	209 (53.5)	273 (41.2)	74 (75.5)	169 (80.1)	
***Former***	269 (21.2)	87 (22.3)	165 (24.9)	11 (11.2)	27 (12.8)	
***Active***	309 (24.3)	95 (24.2)	222 (33.5)	13 (13.3)	15 (7.1)	
**Disease duration at surgery (years), *median (IQR)***	5.9 (1.8 - 11.5)	4.3 (0.9 - 9.3)	6.2 (2.1 - 11.9)	6.5 (2.7 - 14.0)	7.1 (3.1 - 14.2)	.049^a–c^ ^,^ ^e^
**Prior Crohn’s disease medication at surgery, *n (%)***	1068 (82.3)	331 (82.5)	561 (82.5)	70 (68.0)	170 (76.6)	.006^b^ ^,^ ^d^ ^,^ ^e^
***Immunomodulators***	846 (65.4)	279 (69.6)	444 (67.0)	61 (59.2)	122 (55.0)	.045^b^ ^,^ ^c^ ^,^ ^e^
***Biologicals***	895 (69.0)	275 (68.6)	486 (73.3)	47 (45.6)	143 (64.4)	.011^b^ ^,^ ^d–f^
***Anti-TNF agents (IFX, ADA, CTZ, GOL)***	879 (67.8)	273 (68.1)	479 (72.2)	44 (42.7)	139 (62.6)	.007^b^ ^,^ ^d^ ^,^ ^e^ ^,^ ^f^
***Vedolizumab***	98 (7.6)	42 (10.5)	35 (5.3)	7 (6.8)	18 (8.1)	.002^a^
***Ustekinumab***	192 (14.8)	80 (20.0)	88 (13.1)	6 (5.8)	29 (13.1)	.050^a–d^ ^,^ ^f^
***Other biologicals or advanced therapies***	11 (0.8)	7 (1.7)	4 (0.7)	–	–	–
**Lines of preoperative biologicals, *n (%)***						.002^a^ ^,^ ^b^ ^,^ ^e^ ^,^ ^f^
***None***	402 (31.0)	126 (31.4)	178 (26.8)	56 (54.4)	79 (35.6)	
***1***	559 (43.1)	138 (34.4)	335 (52.0)	30 (29.1)	86 (38.7)	
***2***	213 (16.4)	84 (20.9)	89 (13.4)	14 (13.6)	35 (15.8)	
***≥3***	123 (123)	53 (13.2)	51 (7.7)	3 (2.9)	22 (9.9)	
** Age at surgery (*Montreal classification), n (%)***						.014^a–e^
***A1: <17 years***	225 (17.7)	51 (13.5)	89 (13.5)	32 (31.1)	62 (28.4)	
***A2: 17–40 years***	886 (68.2)	254 (67.0)	487 (74.1)	63 (61.2)	129 (59.2)	
***A3: >40 years***	179 (14.1)	74 (19.5)	81 (12.3)	8 (7.8)	27 (12.4)	
**Disease location at surgery (*Montreal classification), n (%)***						.003^c^ ^,^ ^e^
***L1: ileal/ileocecal***	788 (62.9)	245 (64.6)	428 (65.6)	59 (60.2)	114 (54.3)	
***L3: ileocolic***	464 (37.1)	134 (35.4)	195 (33.4)	39 (39.8)	96 (45.7)	
**Disease behaviour at surgery (*Montreal classification), n (%)***						.022^a–c^ ^,^ ^d^ ^,^ ^e^
***B1: non-stricturing, non-penetrating***	117 (9.2)	82 (21.8)	45 (6.8)	0	7 (3.2)	
***B2: stricturing***	559 (43.9)	167 (44.3)	299 (45.4)	48 (46.6)	84 (38.0)	
***B3: penetrating***	596 (46.9)	128 (34.0)	314 (47.7)	55 (53.4)	130 (58.8)	
***+p: perianal disease***	242 (19.2)	55 (14.6)	136 (20.7)	22 (21.6)	49 (23.0)	.026^a^ ^,^ ^c^
High-risk patients**	846 (65.2)	214 (53.4)	479 (72.2)	69 (67.0)	149 (67.1)	.013^a–c^
Number of risk factors**						.009^a^ ^,^ ^c^
***None***	451 (34.8)	187 (46.6)	184 (27.8)	34 (33.0)	73 (32.9)	
***1***	580 (44.7)	156 (38.9)	310 (46.8)	51 (49.5)	107 (48.2)	
***2***	231 (17.8)	52 (13.0)	145 (21.9)	15 (14.6)	39 (17.6)	
***≥3***	35 (2.7)	6 (1.5)	24 (3.6)	3 (2.9	3 (1.4)	

Abbreviations: ADA, adalimumab; CTZ, certolizumab; GOL, golimumab; IFX, infliximab; IQR, interquartile range; TNF, tumour necrosis factors; NS, non-significant (*P* > .05). In case of missing data, valid data are presented.

* P-values for comparison between the four involved countries;

** based on the clinical risk factors in the European guidelines (*active smoking, prior small bowel resection, penetrating disease and/or perianal disease*).

a Netherlands (NL) vs France (FR).

b NL vs Canada (CAN).

c NL vs United States of America [USA].

d FR vs CAN.

e FR. vs USA.

f CAN vs USA (only P-values ≤ .050 are shown; significant P-value is the “highest” value of all comparisons indicating that the P-value is equal or less to the presented P-values).^9^

### 3.2 Patient demographics and disease characteristics at surgery—re-resections

Demographics and clinical characteristics for patients who underwent a re-resection (*n* = 372; 21%) are presented in Table 2 (comparative outcomes are presented in Tables S2a-2f). In contrast to the primary resection group, no significant differences in male sex were observed among patient who underwent a re-resection (NL 51%, FR 47%, CAN 51%, USA 58%). The median age at surgery in the Dutch cohort was significantly higher (49 years) compared to the Canadian cohort (35 years) and the American cohort (42 years; *P* ≤ .041). Significant differences in smoking status were observed, with higher rates of active and former smokers in the European cohorts as compared to the Canadian and American cohorts (*P* ≤ .002). Disease duration at the time of re-resection was similar across cohorts. Rates of preoperative biologicals, including anti-TNF, vedolizumab and ustekinumab, were predominantly higher in the European cohorts without reaching significance in all comparative analyses.

**Table 2.** Baseline characteristics of patients who underwent a re-resection in the study cohort and per country.

Variables	Study cohort (*n* = 372)	Dutch cohort (*n* = 95)	French cohort (*n* = 146)	Canadian cohort (*n* = 45)	American cohort (*n* = 86)	*P*-value*
**Male sex, *n (%)***	179 (52.2)	48 (50.5)	69 (47.3)	23 (51.1)	50 (58.1)	NS
**Age at surgery (years), *median (IQR)***	43.4 (32.5 - 55.0)	48.6 (34.1 - 62.3)	43.5 (33.7 - 53.4)	34.5 (28.2 - 46.5)	42.2 (30.4 - 54.9)	.041^b–d^ ^,^ ^f^
**Smoking status at surgery, *n (%)***						.002^b^ ^,^ ^c^ ^,^ ^d^ ^,^ ^e^
***Never***	166 (49.4)	39 (42.4)	49 (33.8)	35 (77.8)	55 (67.0)	
***Former***	89 (26.5)	26 (28.3)	46 (31.7)	4 (8.9)	18 (22.0)	
***Active***	81 (24.1)	27 (29.3)	50 (34.5)	6 (13.3)	9 (11.0)	
**Disease duration at surgery (years), *median (IQR)***	15.8 (9.8 - 25.0)	15.1 (9.5 - 24.5)	17.3 (11.3 - 24.9)	14.5 (6.9 - 24.1)	15.7 (8.4 - 25.5)	NS
**Prior Crohn’s disease medication at surgery, *n (%)***	290 (84.5)	84 (88.4)	138 (94.5)	34 (75.6)	73 (84.9)	.013^d^ ^,^ ^e^
***Immunomodulators***	247 (72.2)	73 (76.8)	124 (84.9)	22 (48.9)	54 (62.8)	.039^b–e^
***Biologicals***	255 (74.3)	72 (75.8)	118 (81.8)	31 (68.9)	55 (64.0)	.004^e^
***Anti-TNF agents (IFX, ADA, CTZ, GOL)***	246 (71.7)	72 (75.8)	113 (7.4)	28 (62.2)	53 (61.6)	.040^c–e^
***Vedolizumab***	46 (14.0)	22 (23.2)	17 (11.6)	3 (6.7)	10 (11.6)	.042^a–c^
***Ustekinumab***	66 (19.2)	27 (28.4)	28 (19.2)	5 (11.1)	10 (11.6)	.023^b^ ^,^ ^c^
***Other biologicals or advanced therapies***	5 (1.5)	3 (3.2)	2 (1.7)	−	−	−
**Lines of preoperative biologicals, *n (%)***						.005^a^ ^,^ ^b^ ^,^ ^e^
***None***	96 (28.0)	23 (24.2)	28 (19.2)	14 (31.1)	32 (37.2)	
***1***	138 (40.2)	24 (25.3)	75 (51.4)	21 (46.7)	26 (30.2)	
***2***	53 (15.5)	19 (20.0)	31 (21.1)	7 (15.6)	14 (16.3)	
***≥ 3***	56 (16.3)	29 (30.5)	12 (8.2)	3 (6.7)	14 (16.3)	
** Age at diagnosis (*Montreal classification), n (%)***						.034^b–e^
***A1: <17 years***	70 (20.7)	13 (14.5)	19 (13.0)	17 (37.8)	25 (29.1)	
***A2: 17–40 years***	217 (64.2)	57 (63.3)	108 (74.0)	24 (53.3)	50 (58.1)	
***A3: >40 years***	51 (15.1)	20 (22.2)	19 (13.0)	4 (8.9)	11 (12.8)	
**Disease location at surgery (*Montreal classification), n (%)***						NS
***L1: ileal/ileocecal***	186 (56.5)	55 (61.1)	84 (58.3)	26 (59.1)	38 (48.1)	
***L3: ileocolic***	143 (43.5)	35 (38.9)	60 (41.7)	18 (40.9)	41 (51.9)	
**Disease behaviour at surgery (*Montreal classification), n (%)***						.048^d^ ^,^ ^e^
***B1: non-stricturing,non-penetrating***	9 (2.7)	4 (4.4)	6 (4.2)	0	2 (2.3)	
***B2: stricturing***	172 (51.0)	49 (54.4)	85 (59.0)	18 (40.0)	38 (44.2)	
***B3: penetrating***	156 (46.3)	37 (41.1)	53 (36.8)	27 (60.0)	46 (53.5)	
***+p: perianal disease***	88 (26.30	18 (20.0)	47 (32.2)	12 (27.3)	21 (25.3)	.039^a^

Abbreviations: ADA, adalimumab; CTZ, certolizumab; GOL, golimumab; IFX, infliximab; IQR, interquartile range; TNF, tumour necrosis factors; NS, non-significant (P > .05). In case of missing data, valid data are presented.

* P-values for comparison between the four involved countries.

a Netherlands (NL) vs. France (FR).

b NL vs Canada (CAN).

c NL vs United States of America [USA].

d FR vs CAN.

e FR. vs USA.

f CAN vs USA (only P-values ≤0.050 are shown; significant P-value is the “highest” value of all comparisons indicating that the P-value is equal or less to the presented P-values); *as a re-resection is considered as risk factor in the European guidelines, data on high-risk patients or number of risk factors were not compared*.^9^

### 3.3 Postoperative management–primary resections

Among patients who underwent a primary resection, postoperative prophylactic medication was administered to 565/1389 patients (41%; Table 3;  Table S3a-f). In these patients, an anti-TNF agent, ustekinumab, an immunomodulator, vedolizumab, or risankizumab was the treatment of choice in 52% (*n* = 293), 21% (*n* = 121), 21% (*n* = 121), 5% (*n* = 29), and 0.1% (*n* = 1). Postoperative prophylactic medication was used in 28% (*n* = 112) of the Dutch cohort, 48% (*n* = 318) of the French cohort, 28% (*n* = 29) of the Canadian cohort and 48% (*n* = 106) of the American cohort. The rate of postoperative prophylactic medication use was significantly higher in FR and USA as compared to the NL and CAN (*P* ≤ .001).

**Table 3.** Postoperative management and endoscopic outcomes in patients who underwent primary resection per study cohort and per country.

Variables	Study cohort (*n* = 1,389)	Dutch cohort (*n* = 401)	French cohort (*n* = 663)	Canadian cohort (*n* = 103)	American cohort (*n* = 222)	*P*-value*
**Use of postoperative prophylactic medication (<12 weeks), *n (%)***	565 (40.7)	112 (27.9)	318 (48.0)	29 (28.2)	106 (47.7)	<.001^a^ ^,^ ^c^ ^,^ ^d^ ^,^ ^e^
***Immunomodulator (thiopurines or methotrexate)***	121 (21.4)	21 (18.8)	70 (22.0)	5 (17.2)	25 (23.6)	NS
***Anti-TNF agents***	293 (51.8)	58 (51.8)	161 (50.6)	18 (62.1)	56 (52.8)	NS
***Vedolizumab***	29 (5.1)	8 (7.1)	16 (5.0)	2 (6.9)	3 (2.8)	NS
***Ustekinumab***	121 (21.4)	24 (21.4)	71 (22.3)	4 (13.8)	22 (20.8)	NS
***Risankizumab***	1 (0.1)	1 (0.2)	–	–	–	–
**Use of postoperative prophylactic medication in high-risk patients, *n (%)***	378 (44.7)	65 (30.4)	249 (52.0)	20 (29.0)	71 (47.7)	.009^a^ ^,^ ^c^ ^,^ ^d^ ^,^ ^f^
One year follow-up available**, *n (%)*	1,296 (93.3)	380 (94.8)	622 (93.8)	98 (95.1)	196 (88.3)	**–**
**Endoscopic evaluation performed (<1 year postoperatively)**	1,106 (85.3%)	333 (87.6)	545 (87.6)	87 (88.8)	141 (71.9)	<.001^a^ ^,^ ^c–f^
***Median time to first endoscopy (in months), median (IQR)***	6.5 (5.8 - 7.5)	6.2 (5.5 - 7.2)	6.5 (6.1 - 7.5)	6.2 (4.7 - 7.8)	7.3 (5.7 - 9.3)	–
**Available modified Rutgeerts’ score at first postoperative ileocolonoscopy, *n (%)***	1,080 (97.6)	333 (100)	528 (96.9)	86 (98.8)	135 (95.7)	**–**
***i0***	351 (32.5)	73 (22.1)	148 (28.0)	38 (44.2)	92 (68.1)	**–**
***i1***	193 (17.9)	52 (15.7)	103 (19.5)	23 (26.7)	15 (11.1)	**–**
***i2a***	169 (15.6)	70 (21.1)	87 (16.5)	4 (4.7)	8 (5.9)	**–**
***i2b***	227 (21.0)	101 (30.5)	105 (19.9)	11 (12.8)	10 (7.4)	**–**
***i3***	85 (7.9)	31 (9.4)	38 (7.2)	9 (10.5)	7 (5.2)	**–**
***i4***	55 (5.1)	4 (1.2)	47 (8.9)	1 (1.2)	3 (2.2)	**–**
**Endoscopic postoperative recurrence (≥i2b), *n (%)***	367 (34.0)	136 (41.1)	190 (36.0)	21 (24.4)	20 (14.8)	.036^b–e^
**Severe endoscopic postoperative recurrence (≥i3), *n (%)***	140 (13.0)	35 (10.6)	85 (16.1)	10 (11.6)	10 (7.4)	.023^a^ ^,^ ^e^

Abbreviations: IQR, interquartile range; NS, not significant; TNF, tumour necrosis factors (P-value > .05).

* P-values for comparison between the four involved countries.

** 1 year follow-up may not be available as patients underwent ileocolic (re-) resection <1 year without undergoing the first postoperative ileocolonoscopy prior data collection, were referred to another center postoperatively or other reason.

a Netherlands (NL) vs France (FR).

b NL vs Canada (CAN).

c NL vs United States of America [USA].

d FR vs CAN.

e FR. vs USA.

f CAN vs USA (only P-values ≤ .050 are shown; significant P-value is the “highest” value of all comparisons indicating that the P-value is equal or less to the presented P-values).

### 3.4 Postoperative management—re-resections

204/372 patients (55%) who underwent a re-resection were treated with postoperative prophylactic medication (Table 4;  Table S4a-f). In these patients, an anti-TNF agent, ustekinumab, an immunomodulator, vedolizumab, or risankizumab was the treatment of choice in 51% (*n* = 103), 26% (*n* = 53), 14% (*n* = 28), 9% (*n* = 19), and 0.5% (*n* = 1). Postoperative prophylactic medication was used in 47% (*n* = 45) of the Dutch cohort, 61% (*n* = 89) of the French cohort, 51% (*n* = 23) of the Canadian cohort and 54% (*n* = 47) of the American cohort. Prophylactic medication use was significantly higher in FR and the USA compared to the NL (*P* ≤ .038). Additionally, the use of ustekinumab anti-TNF agents as prophylaxis was significantly lower in the NL (33%) compared to other countries (52%-61%; *P* ≤ .040).

**Table 4.** Postoperative management and endoscopic outcomes in patients who underwent a re-resection per study cohort and per country.

Variables	Study cohort (*n* = 372)	Dutch cohort (*n* = 95)	French cohort (*n* = 146)	Canadian cohort (*n* = 45)	American cohort (*n* = 86)	*P*-value*
**Use of postoperative prophylactic medication (<12 weeks), *n (%)***	204 (54.8)	45 (47.4)	89 (61.0)	23 (51.1)	47 (54.7)	.038^a^ ^,^ ^c^ ^,^ ^f^
***Immunomodulator (thiopurines or methotrexate)***	28 (13.7)	8 (17.8)	10 (11.2)	2 (8.7)	8 (17.0)	NS
***Anti-TNF agents***	103 (50.5)	15 (33.3)	46 (51.7)	14 (60.9)	28 (59.6)	.040^a–c^
***Vedolizumab***	19 (9.3)	6 (13.3)	7 (8.9)	2 (8.7)	4 (8.5)	NS
***Ustekinumab***	53 (26.0)	15 (33.3)	26 (29.2)	5 (21.7)	7 (14.9)	.038^c^
***Risankizumab***	1 (0.5)	1 (1.1)	–	–	–	–
One year follow-up available**, *n (%)*	324 (95.3)	91 (95.8)	138 (94.5)	44 (97.8)	75 (87.2)	**–**
**Endoscopic evaluation performed (<1 year postoperatively)**	265 (81.8)	74 (81.3)	108 (78.3)	39 (88.6)	44 (58.7)	.003^c–f^
***Median time to first endoscopy (in months), median (IQR)***	6.5 (5.8 - 7.7)	6.2 (5.3 - 7.1)	6.5 (6.1 - 7.7)	6.2 (5.2 - 8.5)	6.7 (6.2 - 9.9)	–
						^f^
**Available modified Rutgeerts’ score at first postoperative ileocolonoscopy, *n (%)***	260 (98.1)	73 (98.6)	107 (99.1)	38 (97.4)	42 (95.5)	**–**
***i0***	83 (31.9)	19 (26.0)	29 (27.1)	14 (36.8)	21 (50.0)	**–**
***i1***	42 (16.2)	8 (11.0)	17 (15.9)	10 (26.3)	7 (16.7)	**–**
***i2a***	33 (12.7)	15 (20.5)	12 (11.2)	4 (10.5)	2 (4.8)	**–**
***i2b***	61 (23.5)	22 (30.1)	25 (23.4)	9 (23.7)	5 (11.9)	**–**
***i3***	23 (8.8)	7 (9.6)	11 (10.3)	1 (2.6)	4 (9.5)	**–**
***i4***	18 (6.9)	2 (2.7)	13 (12.1)	0	3 (7.1)	**–**
**Endoscopic postoperative recurrence (≥i2b), *n (%)***	102 (39.2)	31 (42.5)	49 (45.8)	10 (26.3)	12 (28.6)	.036^d^
**Severe endoscopic postoperative recurrence (≥i3), *n (%)***	41 (15.8)	9 (12.3)	24 (22.4)	1 (2.6)	7 (16.7)	.037^d^ ^,^ ^f^

Abbreviations: IQR, interquartile range; NS, not significant (P-value >0.05); TNF, tumour necrosis factors.

* P-values for comparison between the four involved countries.

** 1 year follow-up may not be available as patients underwent ileocolic (re-) resection <1 year without undergoing the first postoperative ileocolonoscopy prior data collection, were referred to another center postoperatively or other reason.

a Netherlands (NL) vs France (FR).

b NL vs Canada (CAN).

c NL vs United States of America [USA].

d FR vs CAN.

e FR vs USA.

f CAN vs USA (only P-values ≤ .050 are shown; significant P-value is the “highest” value of all comparisons indicating that the P-value is equal or less to the presented P-values).

### 3.5 Performance of a postoperative ileocolonoscopy and endoscopic outcomes—primary resections

In patients who underwent a primary resection (with 1 year follow-up data available), endoscopic evaluation within 1 year post-surgery was performed in 85% of the patients (*n* = 1106) at a median interval of 6.5 months (interquartile range [IQR] 5.8-7.5; Table 3;  Table S5a-f). The rate of endoscopic evaluation was significantly higher in the NL (88%), FR (88%), and CAN (89%) as compared to the USA cohort (72%, *P* ≤ .001). The distribution of the endoscopic outcomes at the first postoperative ileocolonoscopy for each country is presented in Figure 1A. The overall ePOR and severe ePOR rates was 34% and 13%. Significant higher ePOR rates in the European ­countries (36%-41%) were observed as compared to the North-American countries (15%-24%; *P* ≤ .036). A significant higher severe ePOR rate was observed in FR (16%) as compared to the NL and USA (7%-11%; *P* ≤ .023).

**Figure 1.**
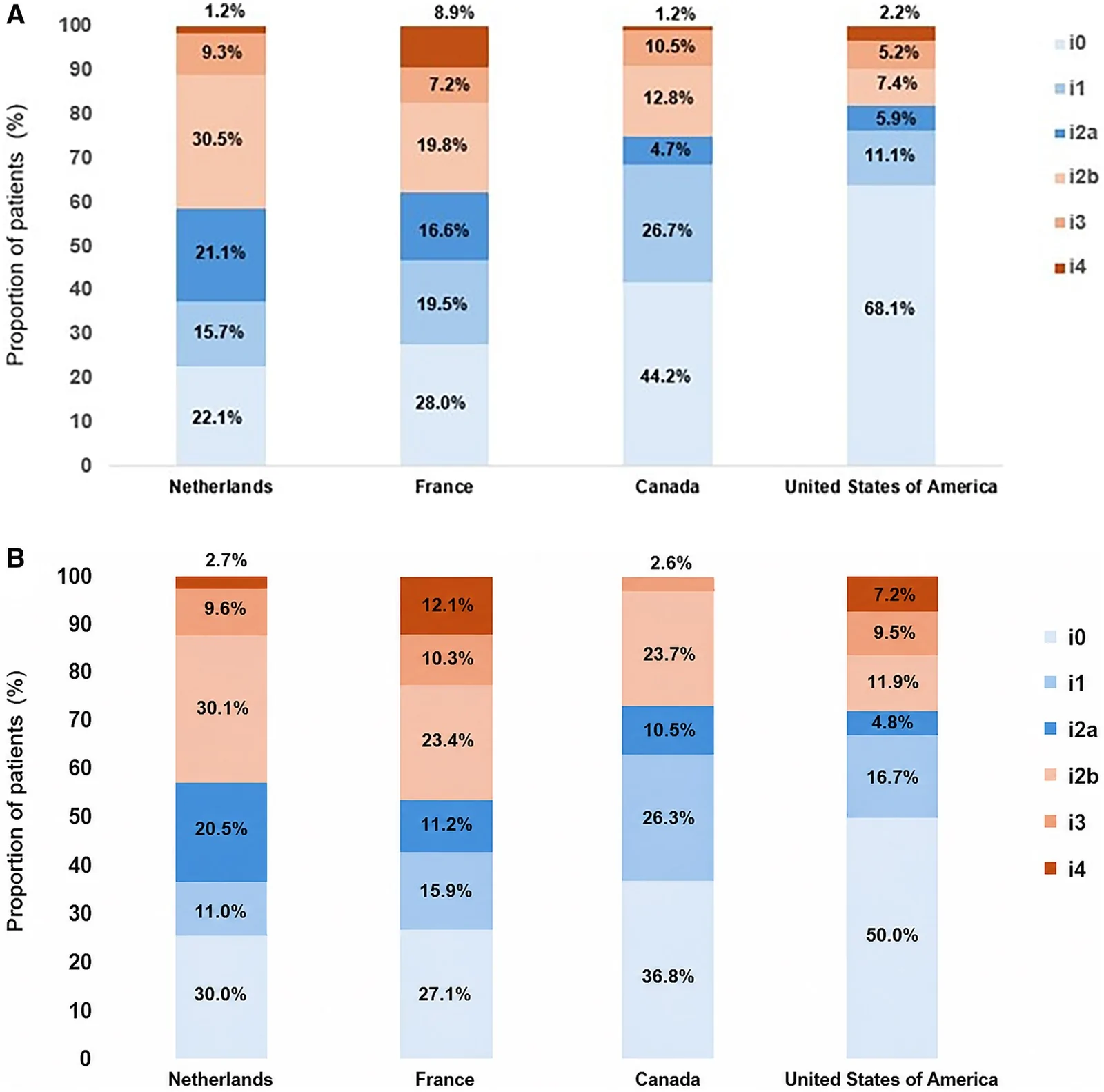
Distribution of the modified Rutgeerts’ score at first postoperative ileocolonoscopy in patients who underwent (A) primary resection and (B) re-resection.

### 3.6 Performance of a postoperative ileocolonoscopy and endoscopic outcomes—re-resections

Within the patients who underwent a re-resection (with 1 year follow-up data available), an endoscopic evaluation within one year post-surgery was performed in 82% (*n* = 265) of the patients after a median interval of 6.5 months (IQR 5.8-7.7; Table 3;  Table S6a-f). The rate of endoscopic evaluation was significantly higher in the NL (81%), FR (78%), and CAN (89%) as compared to the USA cohort (59%, *P* ≤ .003). The distribution of the endoscopic outcomes at the first postoperative ileocolonoscopy for each country is presented in Figure 1B. The overall ePOR and severe ePOR rates were 39% and 16%. Only a significant higher ePOR rate was observed in the French cohort (46%) as compared to the Canadian cohort (26%; *P* = .036). In addition, the severe ePOR rate in the French cohort (22%) was significantly higher as compared to the Canadian (3%) and American cohort (16%; *P* ≤ .037) The distribution of ePOR rates in relationship to (the number of) high-risk features (regardless of primary or re-resection) are presented in Figure S1 and S2.

### 3.7 Explorative analysis regarding the (severe) ePOR risk per country

The explorative analysis, including the countries in the multivariable logistic regression models, showed a comparable risk of developing ePOR in FR (aOR 1.09; 95% CI, 0.81-1.46; *P* = .590) but a decreased (non-significant) risk in CAN (aOR 0.61; 95% CI, 0.37-1.01; *P* = .036) and the USA (aOR 0.57; 95% CI, 0.35-0.93; *P* = .024) as compared to the NL (Table 5). Sensitivity analysis, using FR as a reference, revealed a decreased risk of developing ePOR in CAN (aOR 0.56; 95% CI, 0.35-0.91; *P* = .019) and the USA (aOR 0.53; 95% CI, 0.33-0.83; *P* = .006; Table S7). No difference in ePOR risk was observed between CAN and the USA (aOR 0.94; 95% CI, 0.51-1.72; *P* = .83). For severe ePOR as outcome, only an increased risk was observed in FR as compared to NL (aOR 1.90; 95% CI, 1.24-2.92; *P* = .004; Table 5).

**Table 5.** Multivariable logistic regression models including country in relation to risk of endoscopic postoperative recurrence (modified Rutgeerts’ score ≥ i2b) and severe endoscopic postoperative recurrence (modified Rutgeerts’ score ≥ i3)^a^.

Endoscopic recurrence			
*Country*	aOR	95% CI	*P*-value
**Netherlands**	REF	–	–
**France**	1.09	0.81-1.46	.590
** Canada**	0.61	0.37-1.01	.055
** United States of America**	**0.57**	**0.35-0.93**	**.024**

Severe endoscopic recurrence			
*Country*	aOR	95% CI	*P*-value
**Netherlands**	REF	–	–
** France**	**1.90**	**1.24-2.92**	**.003**
**Canada**	1.03	0.48-2.24	.933
**United States of America**	1.40	0.71-2.75	.328

a Models adjusted for sex, age and disease duration at surgery, smoking status at surgery, prior bowel resection, lines of preoperative biologicals (two or more), disease location and behavior (Montreal L/B/p+), postoperative prophylactic medication, time to ileocolonoscopy and year of surgery.

## 4. Discussion

This large study of prospective cohorts of patients with CD following ICR from four countries in Europe and North America compared preoperative characteristics, postoperative management, and (severe) ePOR rates. Important baseline differences at surgery were identified and most striking within the population who underwent a primary resection. Outcomes of this study provide new insights into geographical differences on preoperative characteristics, postoperative management and postoperative outcomes that must be carefully considered when extrapolating data regarding disease outcomes after surgery in CD. The most important differences between the cohorts concerned active smoking rates, disease duration at time of surgery, biological exposure and disease phenotype. In both primary and re-resection populations, higher ePOR rates were observed in European cohorts than in North American cohorts. These differences between the cohorts remained present after adjustment of potential confounding factors in explorative multivariable regression analyses.

With regards to preoperative characteristics, the indication and timing of surgery vary across cohorts, most remarkably for the primary resection population. A notable finding is the significantly shorter disease duration at surgery in combination with the relatively high rate of patients with uncomplicated disease (Montreal B1) in the Dutch cohort as compared to the other cohorts. This may be explained by the influence of the Dutch LIR! C trial which showed comparable outcomes of laparoscopic ileocecal resection to infliximab for CD patients with limited (<40 cm) and uncomplicated terminal ileitis after failure of immunomodulator therapy.^3^^,^^14^^,^^15^ Based on the outcomes of this pivotal trial, intestinal surgery can be considered as a viable treatment option for uncomplicated terminal ileitis after failing immunomodulator therapy. Another explanation for the variation of disease phenotype at primary ICR may be the higher exposure to IBD medication in the Dutch cohort as compared to the North American cohorts. Another remarkable observation is the higher rates of penetrating disease (Montreal B3) at time of surgery in CAN and the USA which may be linked to variations in preoperative treatment approaches (ie, lower rate of biologicals) as early initiation of biologicals has been associated with lower rates of disease progression.^16^ Furthermore, higher rates of active smokers were reported in the NL and FR. It is unknown whether this higher rate can be attributed to the fact that smoking is underreported in North-America.

With regards to postoperative management, we observed that the vast majority of patients (>80%) in the NL, FR, and CAN underwent an ileocolonoscopy within one year after surgery, whereas only 60%-70% of USA patients underwent an ileocolonoscopy within the proposed time frame (<12 months). The landmark POCER trial demonstrated the superiority of risk-stratified treatment combined with an endoscopy at 6 months, followed by treatment step-up in case recurrence is present, compared to an endoscopy at 18 months postoperatively.^17^ Based on these outcomes, the current guidelines recommend performing an ileocolonoscopy within 1 year. The lower rate of a performed ileocolonoscopy within 12 months postoperatively, within the American cohort, may also be ­influenced by reimbursement policies and healthcare access disparities hampering the performance of postoperative ileocolonoscopy. It is important to note that important data on potential factors influencing postoperative management including ileocolonoscopy within 12 months postoperatively and postoperative prophylaxis in patients at high risk of CD recurrence were not consistently recorded. Factors such as ethnic diversity, patients preferences, shared decision making, access to care (including reimbursement), diet, and socioeconomic status, were not consistently collected across cohorts. Further research is warranted to examine the potential impact of these variables on disease outcomes.

In addition, we observed a low adherence (<50%) to the guidelines regarding the use of postoperative prophylaxis in the high-risk population in the entire cohort, especially in the Dutch and Canadian cohorts in which only 30% of patients received maintenance therapy within 12 weeks postoperatively. Although risk stratification remains a matter of debate, smoking and re-resection are widely accepted as risk factors.^12^ Even in the re-resection population, prophylaxis was as low as 50%-60%. Unfortunately, data on additional risk factors that might have been considered by the treating physicians, such as microscopic inflammation at proximal resection margin, are not available in all cohorts.^7^ The low adherence to guidelines may be explained by limited evidence comparing prophylactic medication versus endoscopy-guided therapy, patients’ preference not to start prophylactic medication and restrictions in reimbursement policies that limit relapse prevention in asymptomatic patients. Further prospective studies are awaited to evaluate machine learning-based models, which may further enhance the probability to adequately predict the ePOR risk as these models can capture complex, non-linear relationships and interactions among predictors without prior assumptions. In addition, new data are required on the outcomes of these 2 different strategies of prophylactic medication versus endoscopy-driven strategy, and the results of the randomized SOPRANO-CD study (NCT05169593) are eagerly awaited.

Geographical differences were also observed in (severe) ePOR rates were observed in our study, namely higher ePOR rates in the European compared to North-American countries, regardless whether had undergone a prior resection. In addition, severe ePOR rates were higher in FR as compared to the North-American cohorts. After adjustment for clinical confounding factors, explorative analyses in the entire cohort (both primary and re-resections) demonstrated an increased risk of developing ePOR in European countries, whereas an increased risk of developing of severe ePOR was observed in FR as compared to the NL. An important observation, in the light of the increased ePOR risk, is the significantly higher proportion of former and active smokers in Europe. Despite adjustment for smoking status, smoking behavior often coexists with other lifestyle factors (such as nutrition, alcohol consumption, and stress) or immunological markers, such as high T cell clonal expansion, along with genetic factors, which may influence the (severe) ePOR risk.^18-21^ However, data on these variables were not collected in these studies. In addition, other factors (eg, ethnic diversity, diet, microbial, and environmental factors) may influence these differences. Further research is warranted to investigate the impact of these factors on disease outcomes in CD.^6^^,^^22^^,^^23^

Despite the comparable study protocols and data collection of 3 large prospective cohort studies, a few limitations of our study should be taken into account. Firstly, data on surgical characteristics, including surgical approach, length of the resection specimen, and type of anastomosis, as well as histopathological factors (eg, inflammation at the resection margins) were not consistently collected across all cohorts. This prevented us from comparing these characteristics. Secondly, it is important to note that important data on potential factors influencing postoperative management including ileocolonoscopy within 12 months postoperatively and postoperative prophylaxis in patients at high risk of CD recurrence were not consistently recorded. Thirdly, despite that all endoscopies were independently reviewed by trained physicians scoring the available videos, images, and/or reports in all separate cohorts, the mRS was assessed based on the available endoscopic videos, images or reports in the Dutch or French cohort, while in the North-American cohort the mRS was assessed only based on the endoscopic reports. In a subset of patients in this cohort, a postoperative endoscopic video was recorded. No statistical difference was observed in ePOR rates between patients with or without endoscopic video suggesting that the difference in endoscopic assessment did not influence the outcomes. Furthermore, the known moderate interobserver agreement for the mRS may also have contributed to differences in ePOR rates.^24^ Finally, this study was conducted in 4 Western countries, which may limit the generalizability of our findings to other regions, such as Asia, South America, Africa, and Oceania.

## 5. Conclusions

Notable differences in perioperative characteristics, postoperative management and outcomes were observed among European and North American patient population, most strikingly in patients who underwent primary resection. In addition, explorative analysis showed a decreased risk of ePOR in North-American countries. Further research is warranted to explore potential explanations for these findings, including patient- and physician-related decision-making, healthcare system factors, and the biological mechanisms underlying postoperative disease recurrence, including the influence of genetic, microbial, and environmental interactions.

## Supplementary Material

jjag137_Supplementary_Data

## Data Availability

The data underlying this article will be shared on reasonable request to the corresponding author.

## Writing assistance

None declared.

RAP-CD study group members are Mariëlle Romberg-Camps; Evelien M. J. Beelen; Sander van der Marel; Frank Hoentjen; Rachel West; Marjolijn Duijvestein; P. W. Jeroen Maljaars; Jarmila D W van der Bilt; Christianne J. Buskens; Laurents P. S. Stassen; Sita Jansen; Nanne K.H. de Boer; Carmen S. Horjus; Laurents P.S. Stassen; Fiona D.M. van Schaik; Bindia J.H. Jharap; and Gerard Dijkstra.

NIDDK Inflammatory Bowel Disease Genetics Consortium members are John Rioux; Dermot McGovern; Richard Duerr; Judy Cho; Steven Brant; and Mark Lazarev. This study was supported by the National Institute of Diabetes and Digestive and Kidney Diseases of the National Institutes of Health under Award Number U01DK062423.

REMIND study group members are Philippe Seksik; Stéphane Nancey; Anthony Buisson; Franck Carbonnel; Laurent Peyrin-Biroulet; Edouard Louis; Xavier Hébuterne; Mélanie Serrero; Arnaud Bourreille; Xavier Treton; Pauline Wils; Mathurin Fumery; Pierre Blanc; Mathieu Uzzan; Nicolas Barnich; and Madeleine Bezault.

## References

[1] Tsai L , Ma C, Dulai PS, et al Contemporary risk of surgery in patients with ulcerative colitis and Crohn’s disease: a meta-analysis of population-based cohorts. Clin Gastroenterol Hepatol. 2021;19:2031-2045 e11. 10.1016/j.cgh.2020.10.039 PMC8934200 33127595

[2] Meima-van Praag EM , Buskens CJ, Hompes R, Bemelman WA. Surgical management of Crohn’s disease: a state of the art review. Int J Colorectal Dis. 2021;36:1133-1145. 10.1007/s00384-021-03857-2 PMC8119249 33528750

[3] Ponsioen CY , de Groof EJ, Eshuis EJ, et al; LIR! C study group. Laparoscopic ileocaecal resection versus infliximab for terminal ileitis in Crohn’s disease: a randomised controlled, open-label, multicentre trial. Lancet Gastroenterol Hepatol. 2017;2:785-792. 10.1016/S2468-1253(17)30248-0 28838644

[4] Arkenbosch JHC , Beelen EMJ, Dijkstra G, et al Prophylactic medication for the prevention of endoscopic recurrence in Crohn’s ­disease: a prospective study based on clinical risk stratification. J Crohns Colitis. 2023;17:221-230. 10.1093/ecco-jcc/jjac128 PMC10024543 36094558

[5] Hammoudi N , Cazals-Hatem D, Auzolle C, et al REMIND Study Group Investigators. Association Between Microscopic Lesions at Ileal Resection Margin and Recurrence After Surgery in Patients With Crohn’s Disease. Clin Gastroenterol Hepatol. 2020;18:141-149 e2. 10.1016/j.cgh.2019.04.045 31042575

[6] Hernández-Rocha C , Walshe M, Birch S, et al Clinical predictors of early and late endoscopic recurrence following ileocolonic resection in Crohn’s disease. J Crohns Colitis. 2024;18:615-627. 10.1093/ecco-jcc/jjad186 PMC11037109 37976264

[7] Bak MTJ , Oudijk L, Ooms AHAG, et al Combined clinical and histopathological risk stratification for prediction of (severe) endoscopic postoperative recurrence in patients with Crohn’s disease after ileocolic resection: results from a prospective multicenter Cohort study. Ann Surg. 2025. 10.1097/SLA.0000000000006749. Epub ahead of print. 40334060

[8] Nguyen GC , Loftus EV Jr, Hirano I, Falck-Ytter Y, Singh S, Sultan S. American Gastroenterological Association Institute guideline on the management of Crohn’s disease after surgical resection. Gastroenterology. 2017;152:271-275. 10.1053/j.gastro.2016.10.038 27840074

[9] Gionchetti P , Dignass A, Danese S, et al 3rd European evidence-based consensus on the diagnosis and management of Crohn’s disease 2016: part 2: surgical management and special situations. J Crohns Colitis. 2017;11:135-149. 10.1093/ecco-jcc/jjw169 27660342

[10] Lamb CA , Kennedy NA, Raine T, et al; IBD guidelines eDelphi consensus group. British Society of Gastroenterology consensus guidelines on the management of inflammatory bowel disease in adults. Gut. 2019;68:s1-s106. 10.1136/gutjnl-2019-318484 PMC6872448 31562236

[11] Rivière P , Bislenghi G, Hammoudi N, et al; 8th Scientific Workshop of the European Crohn’s and Colitis Organisation. Results of the eighth scientific workshop of ECCO: pathophysiology and risk factors of postoperative Crohn’s disease recurrence after an ileocolonic resection. J Crohns Colitis. 2023;17:1557-1568. 10.1093/ecco-jcc/jjad054 37070326

[12] Bak MTJ , Demers K, Hammoudi N, et al Systematic review: patient-related, microbial, surgical, and histopathological risk factors for endoscopic post-operative recurrence in patients with Crohn’s disease. Aliment Pharmacol Ther. 2024;60:310-326. 10.1111/apt.18040 38887827

[13] Højsgaard S , Halekoh U, Yan J. The R Package geepack for generalized estimating equations. J Stat Softw. 2005;15:1-11.

[14] de Groof EJ , Stevens TW, Eshuis EJ, et al; LIR! C study group. Cost-effectiveness of laparoscopic ileocaecal resection versus infliximab treatment of terminal ileitis in Crohn’s disease: the LIR! C Trial. Gut. 2019;68:1774-1780. 10.1136/gutjnl-2018-317539 31233395

[15] Stevens TW , Haasnoot ML, D’Haens GR, et al; LIR! C study group. Laparoscopic ileocaecal resection versus infliximab for terminal ileitis in Crohn’s disease: retrospective long-term follow-up of the LIR! C trial. Lancet Gastroenterol Hepatol. 2020;5:900-907. 10.1016/S2468-1253(20)30117-5 32619413

[16] Revés J , Fernandez-Clotet A, Ordás I, et al Early biological therapy within 12 months of diagnosis leads to higher transmural healing rates in Crohn’s disease. Clin Gastroenterol Hepatol. 2025;23:1194-1203.e2. 10.1016/j.cgh.2024.07.034 39209193

[17] De Cruz P , Kamm MA, Hamilton AL, et al Crohn’s disease management after intestinal resection: a randomised trial. Lancet. 2015;385:1406-1417. 10.1016/S0140-6736(14)61908-5 25542620

[18] Loukola A , Hällfors J, Korhonen T, Kaprio J. Genetics and smoking. Curr Addict Rep. 2014;1:75-82. 10.1007/s40429-013-0006-3 PMC4000030 24778978

[19] Cross SJ , Lotfipour S, Leslie FM. Mechanisms and genetic factors underlying co-use of nicotine and alcohol or other drugs of abuse. Am J Drug Alcohol Abuse. 2017;43:171-185. 10.1080/00952990.2016.1209512 PMC5493323 27532746

[20] Kassel JD , Stroud LR, Paronis CA. Smoking, stress, and negative affect: correlation, causation, and context across stages of smoking. Psychol Bull. 2003;129:270-304. 10.1037/0033-2909.129.2.270 12696841

[21] Allez M , Auzolle C, Ngollo M, et al; REMIND Study Group. T cell clonal expansions in ileal Crohn’s disease are associated with smoking behaviour and postoperative recurrence. Gut. 2019;68:1961-1970. 10.1136/gutjnl-2018-317878 30792246

[22] Bak MTJ , Boland K, Nayeri S, et al Micronutrients are associated with endoscopic postoperative recurrence in Crohn’s disease: a multicenter prospective cohort study in North america. J Crohns Colitis. 2025;20:jjaf148. 10.1093/ecco-jcc/jjaf148 PMC13365150 40833017

[23] Hernández-Rocha C , Turpin W, Borowski K, et al After surgically induced remission, ileal and colonic mucosa-associated microbiota predicts Crohn’s disease recurrence. Clin Gastroenterol Hepatol. 2025;23:612-620 e10. 10.1016/j.cgh.2024.06.022 PMC11979954 38969076

[24] Bak MTJ , Hammoudi N, Allez M, et al; Dutch Initiative on Crohn and Colitis and the RAP-CD study group. Interobserver agreement of current and new proposed endoscopic scores for postoperative recurrence in Crohn’s disease. Gastrointest Endosc. 2024;100:703-709 e4. 10.1016/j.gie.2024.03.011 38462057

