# Supplementary material for: Exploration of international differences in preoperative characteristics, indications, postoperative management and endoscopic recurrence rates in patients with Crohn’s disease following ileocolic (re-)resection: first outcomes from the IMPACT consortium
Source: J Crohns Colitis. 2026 Oct 23;20(10):jjag137. doi: 10.1093/ecco-jcc/jjag137 (PMC13694115; doi:10.1093/ecco-jcc/jjag137)
Supplement: Supplementary file 1 [file jjag137_supplementary_data.docx]

**Supplementary Material**

**Supplementary File 1.** Description of the individual cohorts

*Dutch cohort*

Consecutive patients with Crohn’s disease (CD) who underwent ileocolic (re-)resection (ICR) were identified from an ongoing prospective multicenter, national cohort study (Risk Assessment of Postoperative Recurrence in Crohn’s Disease study; RAP-CD study) in eight academic and seven non-academic hospitals in the Netherlands and endorsed by the Dutch Initiative of Crohn and Colitis (ICC) and the ICC-Surgery (ICC-S). Exclusion criteria comprised (I) indication for ICR other than CD, (II) absence of preoperative ileal disease, (III) presence of gastro-intestinal malignancy in the resection specimen or (IV) the presence of a permanent ileostomy.

A standardized postoperative management algorithm for the start of prophylactic medication was proposed. In this algorithm, all active smokers were strongly advised to quit smoking preoperatively. At ICR, patients were divided into two groups: group 1 (low risk of postoperative recurrence) and group 2 (high risk of postoperative recurrence). Low risk was defined as the absence of risk factors for postoperative recurrence. High risk was defined as the presence of one or more of the following risk factors: active smoking, penetrating disease and/or previous ileocolonic resection. No prophylactic treatment was recommended in group 1, and start of prophylactic therapy after ICR was recommended in group 2. Performance of an ileocolonoscopy at six months postoperatively was encouraged.

*French cohort*

A prospective multicenter cohort study in France and Belgium was initiated by the REMIND (groupe de REcherche sur les Maladies INflammatoires Digestives) group. Inclusion criteria for the cohort were as followed: age greater than 18 years, ileal or ileocolonic CD, and indication of CD‐related intestinal surgery (ileocolonic resection). A postoperative treatment was recommended according to a preestablished algorithm based on the following risk factors: current smoking, previous bowel resection, penetrating phenotype, and active perianal disease. In this algorithm, no treatment or 5‐aminosalycilic acid is proposed to patients with no risk factor; whereas thiopurines, anti‐tumor necrosis factor (TNF) agents or newer biologicals (vedolizumab or ustekinumab) in the case of previous thiopurine failure, is recommended in patients with 1 risk factor; and anti‐TNF therapy (alone or in combination with azathioprine) or newer biologicals in patients with 2 or more risk factors. Performance of an ileocolonoscopy at six months postoperatively was encouraged.

*North-American cohort*

Adult patients with a diagnosis of CD based on standard criteria and undergoing ICR were prospectively identified during the perioperative period across six participating Genetics Research Centers [GRCs] of the NIDDK Inflammatory Bowel Disease Genetics Consortium [IBDGC]. The participant centres comprise Icahn School of Medicine at Mount Sinai, New York; Cedars-Sinai Medical Center, Los Angeles; University of Pittsburgh, Pennsylvania; Johns Hopkins University, Baltimore; Mount Sinai Hospital, Toronto; and University of Montreal, Montreal, along with their affiliated satellite centres. Patients with confirmed Crohn’s disease with ileal involvement based on the surgical pathology review were included. Participants were excluded if they underwent ileal resection with ileal–ileal anastomosis leaving an intact ileocaecal valve, a sub-total or near sub-total colonic resection (i.e. beyond ileal-descending anastomosis), resection with temporary or permanent diverting ileostomy or more than two prior surgeries (i.e. a total of three or more surgeries). The therapeutic approach after surgery, as well as the timing for colonoscopies following resection was determined by the attending gastroenterologist according to standard clinical practice. Patients were mostly identified and recruited in the perioperative period; however, patients could also be enrolled at the first post-surgical endoscopy, as long as that occurred within 18 months after surgery. Only those participants who were amenable to clinical follow-up at the GRC or collaborating site were included.

| Variables | Dutch cohort (n=401) | French cohort (n=663) | p-value |
| --- | --- | --- | --- |
| Male sex, *n(%)* | 161 (40.1) | 326 (49.2) | 0.004 |
| Age at surgery (years), *median (IQR)* | 32.3 (24.8 – 48.0) | 32.1 (25.6 – 42.9) | NS |
| Smoking status at surgery, *n(%)*  *Never*  *Former*  *Active* | 209 (53.5)  87 (22.3)  95 (24.2) | 273 (41.2)  165 (24.9)  222 (33.5) | <0.001 |
| Disease duration at surgery (years), *median (IQR)* | 4.3 (0.9 – 9.3) | 6.2 (2.1 – 11.9) | <0.001 |
| Prior Crohn’s disease medication at surgery, *n(%)*  *Immunomodulators*  *Biologicals*  *Anti-TNF agents (IFX, ADA, CTZ, GOL)*  *Vedolizumab*  *Ustekinumab*  *Other biologicals or advanced therapies* | 331 (82.5)  279 (69.6)  275 (68.6)  273 (68.1)  42 (10.5)  80 (20.0)  7 (1.7) | 561 (82.5)  444 (67.0)  486 (73.3)  479 (72.2)  35 (5.3)  88 (13.1)  4 (0.7) | NS  NS  NS  NS  0.002  0.003  - |
| Lines of preoperative biologicals, *n(%)*  *None*  *1*  *2*  *≥ 3* | 126 (31.4)  138 (34.4)  84 (20.9)  53 (13.2) | 178 (26.8)  335 (52.0)  89 (13.4)  51 (7.7) | <0.001 |
| Age at diagnosis (*Montreal classification), n(%)*  *A1: <17 years*  *A2: 17 – 40 years*  *A3: >40 years* | 51 (13.5)  254 (67.0)  74 (19.5) | 89 (13.5)  487 (74.1)  81 (12.3) | 0.007 |
| Disease location at surgery (*Montreal classification), n(%)*  *L1: ileal/ileocecal*  *L3: ileocolic* | 245 (64.6)  134 (35.4) | 428 (65.6)  195 (33.4) | NS |
| Disease behaviour at surgery (*Montreal classification), n(%)*  *B1: non-stricturing, non-penetrating*  *B2: stricturing*  *B3: penetrating*  *+p: perianal disease* | 82 (21.8)  167 (44.3)  128 (34.0)  55 (14.6) | 45 (6.8)  299 (45.4)  314 (47.7)  136 (20.7) | <0.001  0.026 |
| High-risk patients^*^  Number of risk factors^*^  *None*  *1*  *2*  *≥ 3* | 214 (53.4)  187 (46.6)  156 (38.9)  52 (13.0)  6 (1.5) | 479 (72.2)  184 (27.8)  310 (46.8)  145 (21.9)  24 (3.6) | <0.001  <0.001 |

**Supplementary Table 1.** Comparative analysis of perioperative characteristics between the cohorts in patients who underwent a primary resection (n=1,389)

**Supplementary Table 1a.** Comparative analysis of perioperative characteristics in patients who underwent a primary resection between the Dutch (n=401) and the French cohort (n=663)

**Abbreviations**. IQR = interquartile range; TNF = tumour necrosis factors; IFX = infliximab; ADA = adalimumab; CTZ = certolizumab; GOL = golimumab; NS = non-significant (p>0.05). In case of missing data, valid data are presented.

**^*^** based on the clinical risk factors in the European guidelines (*active smoking, prior small bowel resection, penetrating disease and/or perianal disease*)(8)

**Supplementary Table 1b.** Comparative analysis of perioperative characteristics in patients who underwent a primary resection between the Dutch (n=401) and the Canadian cohort (n=103)

| Variables | Dutch cohort (n=401) | Canadian cohort (n=103) | p-value |
| --- | --- | --- | --- |
| Male sex, *n(%)* | 161 (40.1) | 57 (55.3) | 0.008 |
| Age at surgery (years), *median (IQR)* | 32.3 (24.8 – 48.0) | 31.9 (23.6 – 38.2) | 0.008 |
| Smoking status at surgery, *n(%)*  *Never*  *Former*  *Active* | 209 (53.5)  87 (22.3)  95 (24.2) | 74 (75.5)  11 (11.2)  13 (13.3) | <0.001 |
| Disease duration at surgery (years), *median (IQR)* | 4.3 (0.9 – 9.3) | 6.5 (2.7 – 14.0) | <0.001 |
| Prior Crohn’s disease medication at surgery, *n(%)*  *Immunomodulators*  *Biologicals*  *Anti-TNF agents (IFX, ADA, CTZ, GOL)*  *Vedolizumab*  *Ustekinumab*  *Other biologicals or advanced therapies* | 331 (82.5)  279 (69.6)  275 (68.6)  273 (68.1)  42 (10.5)  80 (20.0)  7 (1.7) | 70 (68.0)  61 (59.2)  47 (45.6)  44 (42.7)  7 (6.8)  6 (5.8)  - | 0.001  0.045  <0.001  <0.001  NS  <0.001  - |
| Lines of preoperative biologicals, *n(%)*  *None*  *1*  *2*  *≥ 3* | 126 (31.4)  138 (34.4)  84 (20.9)  53 (13.2) | 56 (54.4)  30 (29.1)  14 (13.6)  3 (2.9) | <0.001 |
| Age at diagnosis (*Montreal classification), n(%)*  *A1: <17 years*  *A2: 17 – 40 years*  *A3: >40 years* | 51 (13.5)  254 (67.0)  74 (19.5) | 32 (31.1)  63 (61.2)  8 (7.8) | <0.001 |
| Disease location at surgery (*Montreal classification), n(%)*  *L1: ileal/ileocecal*  *L3: ileocolic* | 245 (64.6)  134 (35.4) | 59 (60.2)  39 (39.8) | NS |
| Disease behaviour at surgery (*Montreal classification), n(%)*  *B1: non-stricturing, non-penetrating*  *B2: stricturing*  *B3: penetrating*  *+p: perianal disease* | 82 (21.8)  167 (44.3)  128 (34.0)  55 (14.6) | 0  48 (46.6)  55 (53.4)  22 (21.6) | <0.001  NS |
| High-risk patients^**^  Number of risk factors^**^  *None*  *1*  *2*  *≥ 3* | 214 (53.4)  187 (46.6)  156 (38.9)  52 (13.0)  6 (1.5) | 69 (67.0)  34 (33.0)  51 (49.5)  15 (14.6)  3 (2.9 | 0.013  NS |

**Abbreviations**. IQR = interquartile range; TNF = tumour necrosis factors; IFX = infliximab; ADA = adalimumab; CTZ = certolizumab; GOL = golimumab; NS = non-significant (p>0.05). In case of missing data, valid data are presented.

**^*^** based on the clinical risk factors in the European guidelines (*active smoking, prior small bowel resection, penetrating disease and/or perianal disease*)(8)

**Supplementary Table 1c.** Comparative analysis of perioperative characteristics in patients who underwent a primary resection between the Dutch (n=401) and the American cohort (n=22)

| Variables | Dutch cohort (n=401) | American cohort (n=222) | p-value |
| --- | --- | --- | --- |
| Male sex, *n(%)* | 161 (40.1) | 115 (52.3) | 0.004 |
| Age at surgery (years), *median (IQR)* | 32.3 (24.8 – 48.0) | 29.8 (24.6 – 41.7) | 0.044 |
| Smoking status at surgery, *n(%)*  *Never*  *Former*  *Active* | 209 (53.5)  87 (22.3)  95 (24.2) | 169 (80.1)  27 (12.8)  15 (7.1) | <0.001 |
| Disease duration at surgery (years), *median (IQR)* | 4.3 (0.9 – 9.3) | 7.1 (3.1 – 14.2) | <0.001 |
| Prior Crohn’s disease medication at surgery, *n(%)*  *Immunomodulators*  *Biologicals*  *Anti-TNF agents (IFX, ADA, CTZ, GOL)*  *Vedolizumab*  *Ustekinumab*  *Other biologicals or advanced therapies* | 331 (82.5)  279 (69.6)  275 (68.6)  273 (68.1)  42 (10.5)  80 (20.0)  7 (1.7) | 170 (76.6)  122 (55.0)  143 (64.4)  139 (62.6)  18 (8.1)  29 (13.1)  - | NS  <0.001  NS  NS  NS  0.030  - |
| Lines of preoperative biologicals, *n(%)*  *None*  *1*  *2*  *≥ 3* | 126 (31.4)  138 (34.4)  84 (20.9)  53 (13.2) | 79 (35.6)  86 (38.7)  35 (15.8)  22 (9.9) | NS |
| Age at diagnosis (*Montreal classification), n(%)*  *A1: <17 years*  *A2: 17 – 40 years*  *A3: >40 years* | 51 (13.5)  254 (67.0)  74 (19.5) | 62 (28.4)  129 (59.2)  27 (12.4) | 0.014 |
| Disease location at surgery (*Montreal classification), n(%)*  *L1: ileal/ileocecal*  *L3: ileocolic* | 245 (64.6)  134 (35.4) | 114 (54.3)  96 (45.7) | <0.001 |
| Disease behaviour at surgery (*Montreal classification), n(%)*  *B1: non-stricturing, non-penetrating*  *B2: stricturing*  *B3: penetrating*  *+p: perianal disease* | 82 (21.8)  167 (44.3)  128 (34.0)  55 (14.6) | 7 (3.2)  84 (38.0)  130 (58.8)  49 (23.0) | <0.001  0.010 |
| High-risk patients^**^  Number of risk factors^**^  *None*  *1*  *2*  *≥ 3* | 214 (53.4)  187 (46.6)  156 (38.9)  52 (13.0)  6 (1.5) | 149 (67.1)  73 (32.9)  107 (48.2)  39 (17.6)  3 (1.4) | <0.001  0.009 |

**Abbreviations**. IQR = interquartile range; TNF = tumour necrosis factors; IFX = infliximab; ADA = adalimumab; CTZ = certolizumab; GOL = golimumab; NS = non-significant (p>0.05). In case of missing data, valid data are presented.

**^*^** based on the clinical risk factors in the European guidelines (*active smoking, prior small bowel resection, penetrating disease and/or perianal disease*)(8)

**Supplementary Table 1d.** Comparative analysis of perioperative characteristics in patients who underwent a primary resection between the French (n=663) and the Canadian cohort (n=103)

| Variables | French cohort (n=663) | Canadian cohort (n=103) | p-value |
| --- | --- | --- | --- |
| Male sex, *n(%)* | 326 (49.2) | 57 (55.3) | NS |
| Age at surgery (years), *median (IQR)* | 32.1 (25.6 – 42.9) | 31.9 (23.6 – 38.2) | NS |
| Smoking status at surgery, *n(%)*  *Never*  *Former*  *Active* | 273 (41.2)  165 (24.9)  222 (33.5) | 74 (75.5)  11 (11.2)  13 (13.3) | <0.001 |
| Disease duration at surgery (years), *median (IQR)* | 6.2 (2.1 – 11.9) | 6.5 (2.7 – 14.0) | NS |
| Prior Crohn’s disease medication at surgery, *n(%)*  *Immunomodulators*  *Biologicals*  *Anti-TNF agents (IFX, ADA, CTZ, GOL)*  *Vedolizumab*  *Ustekinumab*  *Other biologicals or advanced therapies* | 561 (82.5)  444 (67.0)  486 (73.3)  479 (72.2)  35 (5.3)  88 (13.1)  4 (0.7) | 70 (68.0)  61 (59.2)  47 (45.6)  44 (42.7)  7 (6.8)  6 (5.8)  - | <0.001  NS  <0.001  <0.001  NS  0.035  - |
| Lines of preoperative biologicals, *n(%)*  *None*  *1*  *2*  *≥ 3* | 178 (26.8)  335 (52.0)  89 (13.4)  51 (7.7) | 56 (54.4)  30 (29.1)  14 (13.6)  3 (2.9) | NS |
| Age at diagnosis (*Montreal classification), n(%)*  *A1: <17 years*  *A2: 17 – 40 years*  *A3: >40 years* | 89 (13.5)  487 (74.1)  81 (12.3) | 32 (31.1)  63 (61.2)  8 (7.8) | <0.001 |
| Disease location at surgery (*Montreal classification), n(%)*  *L1: ileal/ileocecal*  *L3: ileocolic* | 428 (65.6)  195 (33.4) | 59 (60.2)  39 (39.8) | NS |
| Disease behaviour at surgery (*Montreal classification), n(%)*  *B1: non-stricturing, non-penetrating*  *B2: stricturing*  *B3: penetrating*  *+p: perianal disease* | 45 (6.8)  299 (45.4)  314 (47.7)  136 (20.7) | 0  48 (46.6)  55 (53.4)  22 (21.6) | 0.022  NS |
| High-risk patients^**^  Number of risk factors^**^  *None*  *1*  *2*  *≥ 3* | 479 (72.2)  184 (27.8)  310 (46.8)  145 (21.9)  24 (3.6) | 69 (67.0)  34 (33.0)  51 (49.5)  15 (14.6)  3 (2.9 | NS  NS |

**Abbreviations**. IQR = interquartile range; TNF = tumour necrosis factors; IFX = infliximab; ADA = adalimumab; CTZ = certolizumab; GOL = golimumab; NS = non-significant (p>0.05). In case of missing data, valid data are presented.

**^*^** based on the clinical risk factors in the European guidelines (*active smoking, prior small bowel resection, penetrating disease and/or perianal disease*)(8)

**Supplementary Table 1e.** Comparative analysis of perioperative characteristics in patients who underwent a primary resection between the French (n=663) and the American cohort (n=222)

| Variables | French cohort (n=663) | American cohort (n=222) | p-value |
| --- | --- | --- | --- |
| Male sex, *n(%)* | 326 (49.2) | 115 (52.3) | NS |
| Age at surgery (years), *median (IQR)* | 32.1 (25.6 – 42.9) | 29.8 (24.6 – 41.7) | NS |
| Smoking status at surgery, *n(%)*  *Never*  *Former*  *Active* | 273 (41.2)  165 (24.9)  222 (33.5) | 169 (80.1)  27 (12.8)  15 (7.1) | <0.001 |
| Disease duration at surgery (years), *median (IQR)* | 6.2 (2.1 – 11.9) | 7.1 (3.1 – 14.2) | 0.049 |
| Prior Crohn’s disease medication at surgery, *n(%)*  *Immunomodulators*  *Biologicals*  *Anti-TNF agents (IFX, ADA, CTZ, GOL)*  *Vedolizumab*  *Ustekinumab*  *Other biologicals or advanced therapies* | 561 (82.5)  444 (67.0)  486 (73.3)  479 (72.2)  35 (5.3)  88 (13.1)  4 (0.7) | 170 (76.6)  122 (55.0)  143 (64.4)  139 (62.6)  18 (8.1)  29 (13.1)  - | 0.006  0.001  0.011  0.007  NS  NS  - |
| Lines of preoperative biologicals, *n(%)*  *None*  *1*  *2*  *≥ 3* | 178 (26.8)  335 (52.0)  89 (13.4)  51 (7.7) | 79 (35.6)  86 (38.7)  35 (15.8)  22 (9.9) | 0.002 |
| Age at diagnosis (*Montreal classification), n(%)*  *A1: <17 years*  *A2: 17 – 40 years*  *A3: >40 years* | 89 (13.5)  487 (74.1)  81 (12.3) | 62 (28.4)  129 (59.2)  27 (12.4) | <0.001 |
| Disease location at surgery (*Montreal classification), n(%)*  *L1: ileal/ileocecal*  *L3: ileocolic* | 428 (65.6)  195 (33.4) | 114 (54.3)  96 (45.7) | 0.003 |
| Disease behaviour at surgery (*Montreal classification), n(%)*  *B1: non-stricturing, non-penetrating*  *B2: stricturing*  *B3: penetrating*  *+p: perianal disease* | 45 (6.8)  299 (45.4)  314 (47.7)  136 (20.7) | 7 (3.2)  84 (38.0)  130 (58.8)  49 (23.0) | 0.007  NS |
| High-risk patients^**^  Number of risk factors^**^  *None*  *1*  *2*  *≥ 3* | 479 (72.2)  184 (27.8)  310 (46.8)  145 (21.9)  24 (3.6) | 149 (67.1)  73 (32.9)  107 (48.2)  39 (17.6)  3 (1.4) | NS  NS |

**Abbreviations**. IQR = interquartile range; TNF = tumour necrosis factors; IFX = infliximab; ADA = adalimumab; CTZ = certolizumab; GOL = golimumab; NS = non-significant (p>0.05). In case of missing data, valid data are presented.

**^*^** based on the clinical risk factors in the European guidelines (*active smoking, prior small bowel resection, penetrating disease and/or perianal disease*)(8)

**Supplementary Table 1f.** Comparative analysis of perioperative characteristics in patients who underwent a primary resection between the Canadian (n=103) and the American cohort (n=222)

| Variables | Canadian cohort (n=103) | American cohort (n=222) | p-value |
| --- | --- | --- | --- |
| Male sex, *n(%)* | 57 (55.3) | 115 (52.3) | NS |
| Age at surgery (years), *median (IQR)* | 31.9 (23.6 – 38.2) | 29.8 (24.6 – 41.7) | NS |
| Smoking status at surgery, *n(%)*  *Never*  *Former*  *Active* | 74 (75.5)  11 (11.2)  13 (13.3) | 169 (80.1)  27 (12.8)  15 (7.1) | NS |
| Disease duration at surgery (years), *median (IQR)* | 6.5 (2.7 – 14.0) | 7.1 (3.1 – 14.2) | NS |
| Prior Crohn’s disease medication at surgery, *n(%)*  *Immunomodulators*  *Biologicals*  *Anti-TNF agents (IFX, ADA, CTZ, GOL)*  *Vedolizumab*  *Ustekinumab*  *Other biologicals or advanced therapies* | 70 (68.0)  61 (59.2)  47 (45.6)  44 (42.7)  7 (6.8)  6 (5.8)  - | 170 (76.6)  122 (55.0)  143 (64.4)  139 (62.6)  18 (8.1)  29 (13.1)  - | NS  NS  0.001  <0.001  NS  0.050  - |
| Lines of preoperative biologicals, *n(%)*  *None*  *1*  *2*  *≥ 3* | 56 (54.4)  30 (29.1)  14 (13.6)  3 (2.9) | 79 (35.6)  86 (38.7)  35 (15.8)  22 (9.9) | 0.006 |
| Age at diagnosis (*Montreal classification), n(%)*  *A1: <17 years*  *A2: 17 – 40 years*  *A3: >40 years* | 32 (31.1)  63 (61.2)  8 (7.8) | 62 (28.4)  129 (59.2)  27 (12.4) | NS |
| Disease location at surgery (*Montreal classification), n(%)*  *L1: ileal/ileocecal*  *L3: ileocolic* | 59 (60.2)  39 (39.8) | 114 (54.3)  96 (45.7) | NS |
| Disease behaviour at surgery (*Montreal classification), n(%)*  *B1: non-stricturing, non-penetrating*  *B2: stricturing*  *B3: penetrating*  *+p: perianal disease* | 0  48 (46.6)  55 (53.4)  22 (21.6) | 7 (3.2)  84 (38.0)  130 (58.8)  49 (23.0) | NS  NS |
| High-risk patients^**^  Number of risk factors^**^  *None*  *1*  *2*  *≥ 3* | 69 (67.0)  34 (33.0)  51 (49.5)  15 (14.6)  3 (2.9 | 149 (67.1)  73 (32.9)  107 (48.2)  39 (17.6)  3 (1.4) | NS  NS |

**Abbreviations**. IQR = interquartile range; TNF = tumour necrosis factors; IFX = infliximab; ADA = adalimumab; CTZ = certolizumab; GOL = golimumab; NS = non-significant (p>0.05). In case of missing data, valid data are presented.

**^*^** based on the clinical risk factors in the European guidelines (*active smoking, prior small bowel resection, penetrating disease and/or perianal disease*)(8)

| Variables | Dutch cohort (n=95) | French cohort (n=146) | p-value |
| --- | --- | --- | --- |
| Male sex, *n(%)* | 48 (50.5) | 69 (47.3) | NS |
| Age at surgery (years), *median (IQR)* | 48.6 (34.1 – 62.3) | 43.5 (33.7 – 53.4) | NS |
| Smoking status at surgery, *n(%)*  *Never*  *Former*  *Active* | 39 (42.4)  26 (28.3)  27 (29.3) | 49 (33.8)  46 (31.7)  50 (34.5) | NS |
| Disease duration at surgery (years), *median (IQR)* | 15.1 (9.5 – 24.5) | 17.3 (11.3 – 24.9) | NS |
| Prior Crohn’s disease medication at surgery, *n(%)*  *Immunomodulators*  *Biologicals*  *Anti-TNF agents (IFX, ADA, CTZ, GOL)*  *Vedolizumab*  *Ustekinumab*  *Other biologicals or advanced therapies* | 84 (88.4)  73 (76.8)  72 (75.8)  72 (75.8)  22 (23.2)  27 (28.4)  3 (3.2) | 138 (94.5)  124 (84.9)  118 (81.8)  113 (7.4)  17 (11.6)  28 (19.2)  2 (1.7) | NS NS  NS  NS  0.018  NS  - |
| Lines of preoperative biologicals, *n(%)*  *None*  *1*  *2*  *≥ 3* | 23 (24.2)  24 (25.3)  19 (20.0)  29 (30.5) | 28 (19.2)  75 (51.4)  31 (21.1)  12 (8.2) | <0.001 |
| Age at diagnosis (*Montreal classification), n(%)*  *A1: <17 years*  *A2: 17 – 40 years*  *A3: >40 years* | 13 (14.5)  57 (63.3)  20 (22.2) | 19 (13.0)  108 (74.0)  19 (13.0) | NS |
| Disease location at surgery (*Montreal classification), n(%)*  *L1: ileal/ileocecal*  *L3: ileocolic* | 55 (61.1)  35 (38.9) | 84 (58.3)  60 (41.7) | NS |
| Disease behaviour at surgery (*Montreal classification), n(%)*  *B1: non-stricturing, non-penetrating*  *B2: stricturing*  *B3: penetrating*  *+p: perianal disease* | 4 (4.4)  49 (54.4)  37 (41.1)  18 (20.0) | 6 (4.2)  85 (59.0)  53 (36.8)  47 (32.2) | NS  0.039 |

**Supplementary Table 2.** Comparative analysis of perioperative characteristics between the cohorts in patients who underwent a re-resection (n=372)

**Supplementary Table 2a.** Comparative analysis of perioperative characteristics in patients who underwent a re-resection between the Dutch (n=95) and the French cohort (n=146)

**Abbreviations**. IQR = interquartile range; TNF = tumour necrosis factors; IFX = infliximab; ADA = adalimumab; CTZ = certolizumab; GOL = golimumab; NS = non-significant (p>0.05). In case of missing data, valid data are presented.

**Supplementary Table 2b.** Comparative analysis of perioperative characteristics in patients who underwent a re-resection between the Dutch (n=95) and the Canadian cohort (n=45)

| Variables | Dutch cohort (n=95) | Canadian cohort (n=45) | p-value |
| --- | --- | --- | --- |
| Male sex, *n(%)* | 48 (50.5) | 23 (51.1) | NS |
| Age at surgery (years), *median (IQR)* | 48.6 (34.1 – 62.3) | 34.5 (28.2 – 46.5) | <0.001 |
| Smoking status at surgery, *n(%)*  *Never*  *Former*  *Active* | 39 (42.4)  26 (28.3)  27 (29.3) | 35 (77.8)  4 (8.9)  6 (13.3) | <0.001 |
| Disease duration at surgery (years), *median (IQR)* | 15.1 (9.5 – 24.5) | 14.5 (6.9 – 24.1) | NS |
| Prior Crohn’s disease medication at surgery, *n(%)*  *Immunomodulators*  *Biologicals*  *Anti-TNF agents (IFX, ADA, CTZ, GOL)*  *Vedolizumab*  *Ustekinumab*  *Other biologicals or advanced therapies* | 84 (88.4)  73 (76.8)  72 (75.8)  72 (75.8)  22 (23.2)  27 (28.4)  3 (3.2) | 34 (75.6)  22 (48.9)  31 (68.9)  28 (62.2)  3 (6.7)  5 (11.1)  - | NS  <0.001  NS  NS  0.017  0.023  - |
| Lines of preoperative biologicals, *n(%)*  *None*  *1*  *2*  *≥ 3* | 23 (24.2)  24 (25.3)  19 (20.0)  29 (30.5) | 14 (31.1)  21 (46.7)  7 (15.6)  3 (6.7) | 0.005 |
| Age at diagnosis (*Montreal classification), n(%)*  *A1: <17 years*  *A2: 17 – 40 years*  *A3: >40 years* | 13 (14.5)  57 (63.3)  20 (22.2) | 17 (37.8)  24 (53.3)  4 (8.9) | 0.004 |
| Disease location at surgery (*Montreal classification), n(%)*  *L1: ileal/ileocecal*  *L3: ileocolic* | 55 (61.1)  35 (38.9) | 26 (59.1)  18 (40.9) | NS |
| Disease behaviour at surgery (*Montreal classification), n(%)*  *B1: non-stricturing, non-penetrating*  *B2: stricturing*  *B3: penetrating*  *+p: perianal disease* | 4 (4.4)  49 (54.4)  37 (41.1)  18 (20.0) | 0  18 (40.0)  27 (60.0)  12 (27.3) | NS  NS |

**Abbreviations**. IQR = interquartile range; TNF = tumour necrosis factors; IFX = infliximab; ADA = adalimumab; CTZ = certolizumab; GOL = golimumab; NS = non-significant (p>0.05). In case of missing data, valid data are presented.

**Supplementary Table 2c.** Comparative analysis of perioperative characteristics in patients who underwent a re-resection between the Dutch (n=95) and the American cohort (n=86)

| Variables | Dutch cohort (n=95) | American cohort (n=86) | p-value |
| --- | --- | --- | --- |
| Male sex, *n(%)* | 48 (50.5) | 50 (58.1) | NS |
| Age at surgery (years), *median (IQR)* | 48.6 (34.1 – 62.3) | 42.2 (30.4 – 54.9) | 0.041 |
| Smoking status at surgery, *n(%)*  *Never*  *Former*  *Active* | 39 (42.4)  26 (28.3)  27 (29.3) | 55 (67.0)  18 (22.0)  9 (11.0) | <0.001 |
| Disease duration at surgery (years), *median (IQR)* | 15.1 (9.5 – 24.5) | 15.7 (8.4 – 25.5) | NS |
| Prior Crohn’s disease medication at surgery, *n(%)*  *Immunomodulators*  *Biologicals*  *Anti-TNF agents (IFX, ADA, CTZ, GOL)*  *Vedolizumab*  *Ustekinumab*  *Other biologicals or advanced therapies* | 84 (88.4)  73 (76.8)  72 (75.8)  72 (75.8)  22 (23.2)  27 (28.4)  3 (3.2) | 73 (84.9)  54 (62.8)  55 (64.0)  53 (61.6)  10 (11.6)  10 (11.6)  - | NS  0.039  NS  0.040  0.042  0.005  - |
| Lines of preoperative biologicals, *n(%)*  *None*  *1*  *2*  *≥ 3* | 23 (24.2)  24 (25.3)  19 (20.0)  29 (30.5) | 32 (37.2)  26 (30.2)  14 (16.3)  14 (16.3) | NS |
| Age at diagnosis (*Montreal classification), n(%)*  *A1: <17 years*  *A2: 17 – 40 years*  *A3: >40 years* | 13 (14.5)  57 (63.3)  20 (22.2) | 25 (29.1)  50 (58.1)  11 (12.8) | 0.034 |
| Disease location at surgery (*Montreal classification), n(%)*  *L1: ileal/ileocecal*  *L3: ileocolic* | 55 (61.1)  35 (38.9) | 38 (48.1)  41 (51.9) | NS |
| Disease behaviour at surgery (*Montreal classification), n(%)*  *B1: non-stricturing, non-penetrating*  *B2: stricturing*  *B3: penetrating*  *+p: perianal disease* | 4 (4.4)  49 (54.4)  37 (41.1)  18 (20.0) | 2 (2.3)  38 (44.2)  46 (53.5)  21 (25.3) | NS  NS |

**Abbreviations**. IQR = interquartile range; TNF = tumour necrosis factors; IFX = infliximab; ADA = adalimumab; CTZ = certolizumab; GOL = golimumab; NS = non-significant (p>0.05). In case of missing data, valid data are presented.

**Supplementary Table 2d.** Comparative analysis of perioperative characteristics in patients who underwent a re-resection between the French (n=146) and the Canadian cohort (n=45)

**Abbreviations**. IQR = interquartile range; TNF = tumour necrosis factors; IFX = infliximab; ADA = adalimumab; CTZ = certolizumab; GOL = golimumab; NS = non-significant (p>0.05). In case of missing data, valid data are presented.

|  | French cohort (n=146) | Canadian cohort (n=45) | p-value |
| --- | --- | --- | --- |
| Male sex, *n(%)* | 69 (47.3) | 23 (51.1) | NS |
| Age at surgery (years), *median (IQR)* | 43.5 (33.7 – 53.4) | 34.5 (28.2 – 46.5) | <0.001 |
| Smoking status at surgery, *n(%)*  *Never*  *Former*  *Active* | 49 (33.8)  46 (31.7)  50 (34.5) | 35 (77.8)  4 (8.9)  6 (13.3) | <0.001 |
| Disease duration at surgery (years), *median (IQR)* | 17.3 (11.3 – 24.9) | 14.5 (6.9 – 24.1) | NS |
| Prior Crohn’s disease medication at surgery, *n(%)*  *Immunomodulators*  *Biologicals*  *Anti-TNF agents (IFX, ADA, CTZ, GOL)*  *Vedolizumab*  *Ustekinumab*  *Other biologicals or advanced therapies* | 138 (94.5)  124 (84.9)  118 (81.8)  113 (7.4)  17 (11.6)  28 (19.2)  2 (1.7) | 34 (75.6)  22 (48.9)  31 (68.9)  28 (62.2)  3 (6.7)  5 (11.1)  - | <0.001  <0.001  NS  0.043  NS  NS  - |
| Lines of preoperative biologicals, *n(%)*  *None*  *1*  *2*  *≥ 3* | 28 (19.2)  75 (51.4)  31 (21.1)  12 (8.2) | 14 (31.1)  21 (46.7)  7 (15.6)  3 (6.7) | NS |
| Age at diagnosis (*Montreal classification), n(%)*  *A1: <17 years*  *A2: 17 – 40 years*  *A3: >40 years* | 19 (13.0)  108 (74.0)  19 (13.0) | 17 (37.8)  24 (53.3)  4 (8.9) | 0.001 |
| Disease location at surgery (*Montreal classification), n(%)*  *L1: ileal/ileocecal*  *L3: ileocolic* | 84 (58.3)  60 (41.7) | 26 (59.1)  18 (40.9) | NS |
| Disease behaviour at surgery (*Montreal classification), n(%)*  *B1: non-stricturing, non-penetrating*  *B2: stricturing*  *B3: penetrating*  *+p: perianal disease* | 6 (4.2)  85 (59.0)  53 (36.8)  47 (32.2) | 0  18 (40.0)  27 (60.0)  12 (27.3) | 0.048  NS |

| Variables | French cohort (n=146) | American cohort (n=86) | p-value |
| --- | --- | --- | --- |
| Male sex, *n(%)* | 69 (47.3) | 50 (58.1) | NS |
| Age at surgery (years), *median (IQR)* | 43.5 (33.7 – 53.4) | 42.2 (30.4 – 54.9) | NS |
| Smoking status at surgery, *n(%)*  *Never*  *Former*  *Active* | 49 (33.8)  46 (31.7)  50 (34.5) | 55 (67.0)  18 (22.0)  9 (11.0) | <0.001 |
| Disease duration at surgery (years), *median (IQR)* | 17.3 (11.3 – 24.9) | 15.7 (8.4 – 25.5) | NS |
| Prior Crohn’s disease medication at surgery, *n(%)*  *Immunomodulators*  *Biologicals*  *Anti-TNF agents (IFX, ADA, CTZ, GOL)*  *Vedolizumab*  *Ustekinumab*  *Other biologicals or advanced therapies* | 138 (94.5)  124 (84.9)  118 (81.8)  113 (7.4)  17 (11.6)  28 (19.2)  2 (1.7) | 73 (84.9)  54 (62.8)  55 (64.0)  53 (61.6)  10 (11.6)  10 (11.6)  - | 0.013  <0.001  0.004  0.010  NS  NS  - |
| Lines of preoperative biologicals, *n(%)*  *None*  *1*  *2*  *≥ 3* | 28 (19.2)  75 (51.4)  31 (21.1)  12 (8.2) | 32 (37.2)  26 (30.2)  14 (16.3)  14 (16.3) | 0.001 |
| Age at diagnosis (*Montreal classification), n(%)*  *A1: <17 years*  *A2: 17 – 40 years*  *A3: >40 years* | 19 (13.0)  108 (74.0)  19 (13.0) | 25 (29.1)  50 (58.1)  11 (12.8) | 0.009 |
| Disease location at surgery (*Montreal classification), n(%)*  *L1: ileal/ileocecal*  *L3: ileocolic* | 84 (58.3)  60 (41.7) | 38 (48.1)  41 (51.9) | NS |
| Disease behaviour at surgery (*Montreal classification), n(%)*  *B1: non-stricturing, non-penetrating*  *B2: stricturing*  *B3: penetrating*  *+p: perianal disease* | 6 (4.2)  85 (59.0)  53 (36.8)  47 (32.2) | 2 (2.3)  38 (44.2)  46 (53.5)  21 (25.3) | 0.045  NS |

**Supplementary Table 2e.** Comparative analysis of perioperative characteristics in patients who underwent a re-resection between the French (n=146) and the American cohort (n=86)

**Abbreviations**. IQR = interquartile range; TNF = tumour necrosis factors; IFX = infliximab; ADA = adalimumab; CTZ = certolizumab; GOL = golimumab; NS = non-significant (p>0.05). In case of missing data, valid data are presented.

| Variables | Canadian cohort (n=45) | American cohort (n=86) | p-value |
| --- | --- | --- | --- |
| Male sex, *n(%)* | 23 (51.1) | 50 (58.1) | NS |
| Age at surgery (years), *median (IQR)* | 34.5 (28.2 – 46.5) | 42.2 (30.4 – 54.9) | 0.025 |
| Smoking status at surgery, *n(%)*  *Never*  *Former*  *Active* | 35 (77.8)  4 (8.9)  6 (13.3) | 55 (67.0)  18 (22.0)  9 (11.0) | NS |
| Disease duration at surgery (years), *median (IQR)* | 14.5 (6.9 – 24.1) | 15.7 (8.4 – 25.5) | NS |
| Prior Crohn’s disease medication at surgery, *n(%)*  *Immunomodulators*  *Biologicals*  *Anti-TNF agents (IFX, ADA, CTZ, GOL)*  *Vedolizumab*  *Ustekinumab*  *Other biologicals or advanced therapies* | 34 (75.6)  22 (48.9)  31 (68.9)  28 (62.2)  3 (6.7)  5 (11.1)  - | 73 (84.9)  54 (62.8)  55 (64.0)  53 (61.6)  10 (11.6)  10 (11.6)  - | NS  NS  NS  NS  NS  NS  - |
| Lines of preoperative biologicals, *n(%)*  *None*  *1*  *2*  *≥ 3* | 14 (31.1)  21 (46.7)  7 (15.6)  3 (6.7) | 32 (37.2)  26 (30.2)  14 (16.3)  14 (16.3) | NS |
| Age at diagnosis (*Montreal classification), n(%)*  *A1: <17 years*  *A2: 17 – 40 years*  *A3: >40 years* | 17 (37.8)  24 (53.3)  4 (8.9) | 25 (29.1)  50 (58.1)  11 (12.8) | NS |
| Disease location at surgery (*Montreal classification), n(%)*  *L1: ileal/ileocecal*  *L3: ileocolic* | 26 (59.1)  18 (40.9) | 38 (48.1)  41 (51.9) | NS |
| Disease behaviour at surgery (*Montreal classification), n(%)*  *B1: non-stricturing, non-penetrating*  *B2: stricturing*  *B3: penetrating*  *+p: perianal disease* | 0  18 (40.0)  27 (60.0)  12 (27.3) | 2 (2.3)  38 (44.2)  46 (53.5)  21 (25.3) | NS  NS |

**Supplementary Table 2f.** Comparative analysis of perioperative characteristics in patients who underwent a re-resection between the Canadian (n=45) and the American cohort (n=86)

**Abbreviations**. IQR = interquartile range; TNF = tumour necrosis factors; IFX = infliximab; ADA = adalimumab; CTZ = certolizumab; GOL = golimumab; NS = non-significant (p>0.05). In case of missing data, valid data are presented.

**Supplementary Table 3.** Comparative analysis of postoperative management in patients who underwent primary resection between the cohorts

**Supplementary Table 3a.** Comparative analysis of postoperative management in patients who underwent primary resection between the Dutch (n=401) and the French cohort (n=663)

**Abbreviations**. TNF = tumour necrosis factors; IQR = interquartile range; NS = not significant (p-value >0.05)

| Variables | Dutch cohort (n=401) | French cohort (n=663) | p-value |
| --- | --- | --- | --- |
| Use of postoperative prophylactic medication (<12 weeks), *n(%)*  *Immunomodulator (thiopurines or methotrexate)*  *Anti-TNF agents*  *Vedolizumab*  *Ustekinumab*  *Risankizumab*  Use of postoperative prophylactic medication in high-risk patients, *n(%)* | 112 (27.9)  21 (18.8)  58 (51.8)  8 (7.1)  24 (21.4)  1 (0.2)  65 (30.4) | 318 (48.0)  70 (22.0)  161 (50.6)  16 (5.0)  71 (22.3)  -  249 (52.0) | <0.001  NS  NS  NS  NS  NS  <0.001 |

**Supplementary Table 3b.** Comparative analysis of postoperative management in patients who underwent primary resection between the Dutch (n=401) and the Canadian cohort (n=103)

| Variables | Dutch cohort (n=401) | Canadian cohort (n=103) | p-value |
| --- | --- | --- | --- |
| Use of postoperative prophylactic medication (<12 weeks), *n(%)*  *Immunomodulator (thiopurines or methotrexate)*  *Anti-TNF agents*  *Vedolizumab*  *Ustekinumab*  *Risankizumab*  Use of postoperative prophylactic medication in high-risk patients, *n(%)* | 112 (27.9)  21 (18.8)  58 (51.8)  8 (7.1)  24 (21.4)  1 (0.2)  65 (30.4) | 29 (28.2)  5 (17.2)  18 (62.1)  2 (6.9)  4 (13.8)  -  20 (29.0) | NS  NS  NS  NS  NS  -  NS |

**Abbreviations**. TNF = tumour necrosis factors; IQR = interquartile range; NS = not significant (p-value >0.05)

**Supplementary Table 3c.** Comparative analysis of postoperative management in patients who underwent primary resection between the Dutch (n=401) and American cohort (n=222)

**Abbreviations**. TNF = tumour necrosis factors; IQR = interquartile range; NS = not significant (p-value >0.05)

| Variables | Dutch cohort (n=401) | American cohort (n=222) | p-value |
| --- | --- | --- | --- |
| Use of postoperative prophylactic medication (<12 weeks), *n(%)*  *Immunomodulator (thiopurines or methotrexate)*  *Anti-TNF agents*  *Vedolizumab*  *Ustekinumab*  *Risankizumab*  Use of postoperative prophylactic medication in high-risk patients, *n(%)* | 112 (27.9)  21 (18.8)  58 (51.8)  8 (7.1)  24 (21.4)  1 (0.2)  65 (30.4) | 106 (47.7)  25 (23.6)  56 (52.8)  3 (2.8)  22 (20.8)  -  71 (47.7) | <0.001  NS  NS  NS  NS  -  <0.001 |

| Variables | French cohort (n=663) | Canadian cohort (n=103) | p-value |
| --- | --- | --- | --- |
| Use of postoperative prophylactic medication (<12 weeks), *n(%)*  *Immunomodulator (thiopurines or methotrexate)*  *Anti-TNF agents*  *Vedolizumab*  *Ustekinumab*  *Risankizumab*  Use of postoperative prophylactic medication in high-risk patients, *n(%)* | 318 (48.0)  70 (22.0)  161 (50.6)  16 (5.0)  71 (22.3)  -  249 (52.0) | 29 (28.2)  5 (17.2)  18 (62.1)  2 (6.9)  4 (13.8)  -  20 (29.0) | <0.001  NS  NS  NS  NS  -  <0.001 |

**Supplementary Table 3d.** Comparative analysis of postoperative management in patients who underwent primary resection between the French (n=663) and Canadian cohort (n=103)

**Abbreviations**. TNF = tumour necrosis factors; IQR = interquartile range; NS = not significant (p-value >0.05)

| Variables | French cohort (n=663) | American cohort (n=222) | p-value |
| --- | --- | --- | --- |
| Use of postoperative prophylactic medication (<12 weeks), *n(%)*  *Immunomodulator (thiopurines or methotrexate)*  *Anti-TNF agents*  *Vedolizumab*  *Ustekinumab*  *Risankizumab*  Use of postoperative prophylactic medication in high-risk patients, *n(%)* | 318 (48.0)  70 (22.0)  161 (50.6)  16 (5.0)  71 (22.3)  -  249 (52.0) | 106 (47.7)  25 (23.6)  56 (52.8)  3 (2.8)  22 (20.8)  -  71 (47.7) | NS  NS  NS  NS  NS  -  NS |

**Supplementary Table 3e.** Comparative analysis of postoperative management in patients who underwent primary resection between the French (n=663) and American cohort (n=222)

**Abbreviations**. TNF = tumour necrosis factors; IQR = interquartile range; NS = not significant (p-value >0.05)

**Supplementary Table 3f.** Comparative analysis of postoperative management in patients who underwent primary resection between Canadian cohort (n=103) and the American cohort (n=222)

**Abbreviations**. TNF = tumour necrosis factors; IQR = interquartile range; NS = not significant (p-value >0.05)

| Variables | Canadian cohort (n=103) | American cohort (n=222) | p-value |
| --- | --- | --- | --- |
| Use of postoperative prophylactic medication (<12 weeks), *n(%)*  *Immunomodulator (thiopurines or methotrexate)*  *Anti-TNF agents*  *Vedolizumab*  *Ustekinumab*  *Risankizumab*  Use of postoperative prophylactic medication in high-risk patients, *n(%)* | 29 (28.2)  5 (17.2)  18 (62.1)  2 (6.9)  4 (13.8)  -  20 (29.0) | 106 (47.7)  25 (23.6)  56 (52.8)  3 (2.8)  22 (20.8)  -  71 (47.7) | <0.001  NS  NS  NS  NS  -  0.009 |

**Supplementary Table 4.** Comparative analysis of postoperative management in patients who underwent re-resection between the cohorts

**Supplementary Table 4a.** Comparative analysis of postoperative management in patients who underwent a re-resection between the Dutch (n=95) and the French cohort (n=146)

**Abbreviations**. TNF = tumour necrosis factors; IQR = interquartile range; NS = not significant (p-value >0.05)

| Variables | Dutch cohort (n=95) | French cohort (n=146) | p-value |
| --- | --- | --- | --- |
| Use of postoperative prophylactic medication (<12 weeks), *n(%)*  *Immunomodulator (thiopurines or methotrexate)*  *Anti-TNF agents*  *Vedolizumab*  *Ustekinumab*  *Risankizumab* | 45 (47.4)  8 (17.8)  15 (33.3)  6 (13.3)  15 (33.3)  1 (1.1) | 89 (61.0)  10 (11.2)  46 (51.7)  7 (8.9)  26 (29.2)  - | 0.038  NS  0.040  NS  NS  NS |

**Supplementary Table 4b.** Comparative analysis of postoperative management in patients who underwent a re-resection between the Dutch (n=95) and the Canadian cohort (n=45)

| Variables | Dutch cohort (n=95) | Canadian cohort (n=45) | p-value |
| --- | --- | --- | --- |
| Use of postoperative prophylactic medication (<12 weeks), *n(%)*  *Immunomodulator (thiopurines or methotrexate)*  *Anti-TNF agents*  *Vedolizumab*  *Ustekinumab*  *Risankizumab* | 45 (47.4)  8 (17.8)  15 (33.3)  6 (13.3)  15 (33.3)  1 (1.1) | 23 (51.1)  2 (8.7)  14 (60.9)  2 (8.7)  5 (21.7)  - | NS  NS  0.030  NS  NS  - |

**Abbreviations**. TNF = tumour necrosis factors; IQR = interquartile range; NS = not significant (p-value >0.05)

**Supplementary Table 4c.** Comparative analysis of postoperative management in patients who underwent a re-resection between the Dutch (n=95) and the American cohort (n=86)

| Variables | Dutch cohort (n=95) | American cohort (n=86) | p-value |
| --- | --- | --- | --- |
| Use of postoperative prophylactic medication (<12 weeks), *n(%)*  *Immunomodulator (thiopurines or methotrexate)*  *Anti-TNF agents*  *Vedolizumab*  *Ustekinumab*  *Risankizumab* | 45 (47.4)  8 (17.8)  15 (33.3)  6 (13.3)  15 (33.3)  1 (1.1) | 47 (54.7)  8 (17.0)  28 (59.6)  4 (8.5)  7 (14.9)  - | <0.001  NS  0.012  NS  0.038  - |

**Abbreviations**. TNF = tumour necrosis factors; IQR = interquartile range; NS = not significant (p-value >0.05)

| Variables | French cohort (n=146) | American cohort (n=86) | p-value |
| --- | --- | --- | --- |
| Use of postoperative prophylactic medication (<12 weeks), *n(%)*  *Immunomodulator (thiopurines or methotrexate)*  *Anti-TNF agents*  *Vedolizumab*  *Ustekinumab*  *Risankizumab* | 89 (61.0)  10 (11.2)  46 (51.7)  7 (8.9)  26 (29.2)  - | 47 (54.7)  8 (17.0)  28 (59.6)  4 (8.5)  7 (14.9)  - | NS  NS  NS  NS  NS  - |

| Variables | French cohort (n=146) | Canadian cohort (n=45) | p-value |
| --- | --- | --- | --- |
| Use of postoperative prophylactic medication (<12 weeks), *n(%)*  *Immunomodulator (thiopurines or methotrexate)*  *Anti-TNF agents*  *Vedolizumab*  *Ustekinumab*  *Risankizumab* | 89 (61.0)  10 (11.2)  46 (51.7)  7 (8.9)  26 (29.2)  - | 23 (51.1)  2 (8.7)  14 (60.9)  2 (8.7)  5 (21.7)  - | NS  NS  NS  NS  NS  - |

**Supplementary Table 4d.** Comparative analysis of postoperative management in patients who underwent a re-resection between the French (n=146) and the Canadian cohort (n=45)

**Abbreviations**. TNF = tumour necrosis factors; IQR = interquartile range; NS = not significant (p-value >0.05)

**Supplementary Table 4e.** Comparative analysis of postoperative management in patients who underwent a re-resection between the French (n=146) and the American cohort (n=86)

**Abbreviations**. TNF = tumour necrosis factors; IQR = interquartile range; NS = not significant (p-value >0.05)

**Supplementary Table 4f.** Comparative analysis of postoperative management in patients who underwent a re-resection between the Canadian (n=45) and the American cohort (n=86)

**Abbreviations**. TNF = tumour necrosis factors; IQR = interquartile range; NS = not significant (p-value >0.05)

| Variables | Canadian cohort (n=45) | American cohort (n=86) | p-value |
| --- | --- | --- | --- |
| Use of postoperative prophylactic medication (<12 weeks), *n(%)*  *Immunomodulator (thiopurines or methotrexate)*  *Anti-TNF agents*  *Vedolizumab*  *Ustekinumab*  *Risankizumab* | 23 (51.1)  2 (8.7)  14 (60.9)  2 (8.7)  5 (21.7)  - | 47 (54.7)  8 (17.0)  28 (59.6)  4 (8.5)  7 (14.9)  - | 0.003  NS  NS  NS  NS  - |

**Supplementary Table 5.** Comparative analysis of endoscopic postoperative management and outcomes in patients who underwent primary resection between the cohort (n=1,389)

| Variables | Dutch cohort (n=401) | American cohort (n=222) | p-value |
| --- | --- | --- | --- |
| One year follow-up available^**^,  *n(%)*  Endoscopic evaluation performed (<1 year postoperatively)    *Median time to first endoscopy (in months), median (IQR)*  Available modified Rutgeerts’ score at first postoperative ileocolonoscopy, *n(%)*  *i0*  *i1*  *i2a*  *i2b*  *i3*  *i4*  Endoscopic postoperative recurrence (≥i2b), *n(%)*  Severe endoscopic postoperative recurrence (≥i3), *n(%)* | 380 (94.8)  333 (87.6)  6.2 (5.5 – 7.2)  333 (100)  73 (22.1)  52 (15.7)  70 (21.1)  101 (30.5)  31 (9.4)  4 (1.2)  136 (41.1)  35 (10.6) | 196 (88.3)  141 (71.9)  7.3 (5.7 – 9.3)  135 (95.7)  92 (68.1)  15 (11.1)  8 (5.9)  10 (7.4)  7 (5.2)  3 (2.2)  20 (14.8)  10 (7.4) | **-**  <0.001  -  **-**  **-**  **-**  **-**  **-**  **-**  **-**  <0.001  NS |

**Supplementary Table 5a.** Comparative analysis of endoscopic postoperative management and outcomes in patients who underwent primary resection between the Dutch (n=401) and the French cohort (n=663)

| Variables | Dutch cohort (n=401) | French cohort (n=663) | p-value |
| --- | --- | --- | --- |
| One year follow-up available^**^,  *n(%)*  Endoscopic evaluation performed (<1 year postoperatively)    *Median time to first endoscopy (in months), median (IQR)*  Available modified Rutgeerts’ score at first postoperative ileocolonoscopy, *n(%)*  *i0*  *i1*  *i2a*  *i2b*  *i3*  *i4*  Endoscopic postoperative recurrence (≥i2b), *n(%)*  Severe endoscopic postoperative recurrence (≥i3), *n(%)* | 380 (94.8)  333 (87.6)  6.2 (5.5 – 7.2)  333 (100)  73 (22.1)  52 (15.7)  70 (21.1)  101 (30.5)  31 (9.4)  4 (1.2)  136 (41.1)  35 (10.6) | 622 (93.8)  545 (87.6)  6.5 (6.1 – 7.5)  528 (96.9)  148 (28.0)  103 (19.5)  87 (16.5)  105 (19.9)  38 (7.2)  47 (8.9)  190 (36.0)  85 (16.1) | **-**  **<**0.001  **-**  **-**  **-**  **-**  **-**  **-**  **-**  **-**  NS  0.023 |

**Abbreviations**. NS = not significant (p-value >0.05)

| Variables | Dutch cohort (n=401) | Canadian cohort (n=103) | p-value |
| --- | --- | --- | --- |
| One year follow-up available^**^,  *n(%)*  Endoscopic evaluation performed (<1 year postoperatively)    *Median time to first endoscopy (in months), median (IQR)*  Available modified Rutgeerts’ score at first postoperative ileocolonoscopy, *n(%)*  *i0*  *i1*  *i2a*  *i2b*  *i3*  *i4*  Endoscopic postoperative recurrence (≥i2b), *n(%)*  Severe endoscopic postoperative recurrence (≥i3), *n(%)* | 380 (94.8)  333 (87.6)  6.2 (5.5 – 7.2)  333 (100)  73 (22.1)  52 (15.7)  70 (21.1)  101 (30.5)  31 (9.4)  4 (1.2)  136 (41.1)  35 (10.6) | 98 (95.1)  87 (88.8)  6.2 (4.7 – 7.8)  86 (98.8)  38 (44.2)  23 (26.7)  4 (4.7)  11 (12.8)  9 (10.5)  1 (1.2)  21 (24.4)  10 (11.6) | **-**  NS  **-**  **-**  **-**  **-**  **-**  **-**  **-**  **-**  0.001  NS |

**Supplementary Table 5b.** Comparative analysis of endoscopic postoperative management and outcomes in patients who underwent primary resection between the Dutch (n=401) and the Canadian cohort (n=103)

**Abbreviations**. NS = not significant (p-value >0.05)

**Supplementary Table 5c.** Comparative analysis of endoscopic postoperative management and outcomes in patients who underwent primary resection between the Dutch (n=401) and American cohort (n=222)

**Abbreviations**. NS = not significant (p-value >0.05)

**Supplementary Table 5d.** Comparative analysis of endoscopic postoperative management and outcomes in patients who underwent primary resection between the French (n=663) and Canadian cohort (n=103)

| Variables | French cohort (n=663) | Canadian cohort (n=103) | p-value |
| --- | --- | --- | --- |
| One year follow-up available^**^,  *n(%)*  Endoscopic evaluation performed (<1 year postoperatively)    *Median time to first endoscopy (in months), median (IQR)*  Available modified Rutgeerts’ score at first postoperative ileocolonoscopy, *n(%)*  *i0*  *i1*  *i2a*  *i2b*  *i3*  *i4*  Endoscopic postoperative recurrence (≥i2b), *n(%)*  Severe endoscopic postoperative recurrence (≥i3), *n(%)* | 622 (93.8)  545 (87.6)  6.5 (6.1 – 7.5)  528 (96.9)  148 (28.0)  103 (19.5)  87 (16.5)  105 (19.9)  38 (7.2)  47 (8.9)  190 (36.0)  85 (16.1) | 98 (95.1)  87 (88.8)  6.2 (4.7 – 7.8)  86 (98.8)  38 (44.2)  23 (26.7)  4 (4.7)  11 (12.8)  9 (10.5)  1 (1.2)  21 (24.4)  10 (11.6) | **-**  **<**0.001  -  **-**  **-**  **-**  **-**  **-**  **-**  **-**  0.036  NS |

**Abbreviations**. NS = not significant (p-value >0.05)

**Supplementary Table 5e.** Comparative analysis of endoscopic postoperative management and outcomes in patients who underwent primary resection between the French (n=663) and American cohort (n=222)

| Variables | French cohort (n=663) | American cohort (n=222) | p-value |
| --- | --- | --- | --- |
| One year follow-up available^**^,  *n(%)*  Endoscopic evaluation performed (<1 year postoperatively)    *Median time to first endoscopy (in months), median (IQR)*  Available modified Rutgeerts’ score at first postoperative ileocolonoscopy, *n(%)*  *i0*  *i1*  *i2a*  *i2b*  *i3*  *i4*  Endoscopic postoperative recurrence (≥i2b), *n(%)*  Severe endoscopic postoperative recurrence (≥i3), *n(%)* | 622 (93.8)  545 (87.6)  6.5 (6.1 – 7.5)  528 (96.9)  148 (28.0)  103 (19.5)  87 (16.5)  105 (19.9)  38 (7.2)  47 (8.9)  190 (36.0)  85 (16.1) | 196 (88.3)  141 (71.9)  7.3 (5.7 – 9.3)  135 (95.7)  92 (68.1)  15 (11.1)  8 (5.9)  10 (7.4)  7 (5.2)  3 (2.2)  20 (14.8)  10 (7.4) | **-**  **<**0.001  **-**  **-**  **-**  **-**  **-**  **-**  **-**  **-**  <0.001  0.010 |

**Abbreviations**. NS = not significant (p-value >0.05)

| Variables | Canadian cohort (n=103) | American cohort (n=222) | p-value |
| --- | --- | --- | --- |
| One year follow-up available^**^,  *n(%)*  Endoscopic evaluation performed (<1 year postoperatively)    *Median time to first endoscopy (in months), median (IQR)*  Available modified Rutgeerts’ score at first postoperative ileocolonoscopy, *n(%)*  *i0*  *i1*  *i2a*  *i2b*  *i3*  *i4*  Endoscopic postoperative recurrence (≥i2b), *n(%)*  Severe endoscopic postoperative recurrence (≥i3), *n(%)* | 98 (95.1)  87 (88.8)  6.2 (4.7 – 7.8)  86 (98.8)  38 (44.2)  23 (26.7)  4 (4.7)  11 (12.8)  9 (10.5)  1 (1.2)  21 (24.4)  10 (11.6) | 196 (88.3)  141 (71.9)  7.3 (5.7 – 9.3)  135 (95.7)  92 (68.1)  15 (11.1)  8 (5.9)  10 (7.4)  7 (5.2)  3 (2.2)  20 (14.8)  10 (7.4) | **-**  0.001  **-**  **-**  **-**  **-**  **-**  **-**  **-**  **-**  NS  NS |

**Supplementary Table 5f.** Comparative analysis of endoscopic postoperative management and outcomes in patients who underwent primary resection between Canadian cohort (n=103) and the American cohort (n=222)

**Abbreviations**. NS = not significant (p-value >0.05)

**Supplementary Table 6.** Comparative analysis of endoscopic postoperative management and outcomes in patients who underwent a re-resection between the cohort

| Variables | Dutch cohort (n=95) | Canadian cohort (n=45) | p-value |
| --- | --- | --- | --- |
| One year follow-up available^**^,  *n(%)*  Endoscopic evaluation performed (<1 year postoperatively)    *Median time to first endoscopy (in months), median (IQR)*  Available modified Rutgeerts’ score at first postoperative ileocolonoscopy, *n(%)*  *i0*  *i1*  *i2a*  *i2b*  *i3*  *i4*  Endoscopic postoperative recurrence (≥i2b), *n(%)*  Severe endoscopic postoperative recurrence (≥i3), *n(%)* | 91 (95.8)  74 (81.3)  6.2 (5.3 – 7.1)  73 (98.6)  19 (26.0)  8 (11.0)  15 (20.5)  22 (30.1)  7 (9.6)  2 (2.7)  31 (42.5)  9 (12.3) | 44 (97.8)  39 (88.6)  6.2 (5.2 – 8.5)  38 (97.4)  14 (36.8)  10 (26.3)  4 (10.5)  9 (23.7)  1 (2.6)  0  10 (26.3)  1 (2.6) | **-**  NS  **-**  **-**  **-**  **-**  **-**  **-**  **-**  **-**  NS  NS |

| Variables | Dutch cohort (n=95) | French cohort (n=146) | p-value |
| --- | --- | --- | --- |
| One year follow-up available^**^,  *n(%)*  Endoscopic evaluation performed (<1 year postoperatively)    *Median time to first endoscopy (in months), median (IQR)*  Available modified Rutgeerts’ score at first postoperative ileocolonoscopy, *n(%)*  *i0*  *i1*  *i2a*  *i2b*  *i3*  *i4*  Endoscopic postoperative recurrence (≥i2b), *n(%)*  Severe endoscopic postoperative recurrence (≥i3), *n(%)* | 91 (95.8)  74 (81.3)  6.2 (5.3 – 7.1)  73 (98.6)  19 (26.0)  8 (11.0)  15 (20.5)  22 (30.1)  7 (9.6)  2 (2.7)  31 (42.5)  9 (12.3) | 138 (94.5)  108 (78.3)  6.5 (6.1 – 7.7)  107 (99.1)  29 (27.1)  17 (15.9)  12 (11.2)  25 (23.4)  11 (10.3)  13 (12.1)  49 (45.8)  24 (22.4) | **-**  NS  **-**  **-**  **-**  **-**  **-**  **-**  **-**  **-**  NS  NS |

**Supplementary Table 6a.** Comparative analysis of endoscopic postoperative management and outcomes in patients who underwent a re-resection between the Dutch (n=95) and the French cohort (n=146)

**Abbreviations**. NS = not significant (p-value >0.05)

**Supplementary Table 6b.** Comparative analysis of endoscopic postoperative management and outcomes in patients who underwent a re-resection between the Dutch (n=95) and the Canadian cohort (n=45)

**Abbreviations**. NS = not significant (p-value >0.05)

| Variables | Dutch cohort (n=95) | American cohort (n=86) | p-value |
| --- | --- | --- | --- |
| One year follow-up available^**^,  *n(%)*  Endoscopic evaluation performed (<1 year postoperatively)    *Median time to first endoscopy (in months), median (IQR)*  Available modified Rutgeerts’ score at first postoperative ileocolonoscopy, *n(%)*  *i0*  *i1*  *i2a*  *i2b*  *i3*  *i4*  Endoscopic postoperative recurrence (≥i2b), *n(%)*  Severe endoscopic postoperative recurrence (≥i3), *n(%)* | 91 (95.8)  74 (81.3)  6.2 (5.3 – 7.1)  73 (98.6)  19 (26.0)  8 (11.0)  15 (20.5)  22 (30.1)  7 (9.6)  2 (2.7)  31 (42.5)  9 (12.3) | 75 (87.2)  44 (58.7)  6.7 (6.2 – 9.9)  42 (95.5)  21 (50.0)  7 (16.7)  2 (4.8)  5 (11.9)  4 (9.5)  3 (7.1)  12 (28.6)  7 (16.7) | **-**  0.001  -  **-**  **-**  **-**  **-**  **-**  **-**  **-**  NS  NS |

**Supplementary Table 6c.** Comparative analysis of endoscopic postoperative management and outcomes in patients who underwent a re-resection between the Dutch (n=95) and American cohort (n=86)

**Abbreviations**. NS = not significant (p-value >0.05)

| Variables | French cohort (n=146) | Canadian cohort (n=45) | p-value |
| --- | --- | --- | --- |
| One year follow-up available^**^,  *n(%)*  Endoscopic evaluation performed (<1 year postoperatively)    *Median time to first endoscopy (in months), median (IQR)*  Available modified Rutgeerts’ score at first postoperative ileocolonoscopy, *n(%)*  *i0*  *i1*  *i2a*  *i2b*  *i3*  *i4*  Endoscopic postoperative recurrence (≥i2b), *n(%)*  Severe endoscopic postoperative recurrence (≥i3), *n(%)* | 138 (94.5)  108 (78.3)  6.5 (6.1 – 7.7)  107 (99.1)  29 (27.1)  17 (15.9)  12 (11.2)  25 (23.4)  11 (10.3)  13 (12.1)  49 (45.8)  24 (22.4) | 44 (97.8)  39 (88.6)  6.2 (5.2 – 8.5)  38 (97.4)  14 (36.8)  10 (26.3)  4 (10.5)  9 (23.7)  1 (2.6)  0  10 (26.3)  1 (2.6) | **-**  NS  -  **-**  **-**  **-**  **-**  **-**  **-**  **-**  0.036  0.006 |

**Supplementary Table 6d.** Comparative analysis of endoscopic postoperative management and outcomes in patients who underwent a re-resection between the French (n=146) and Canadian cohort (n=45)

**Abbreviations**. NS = not significant (p-value >0.05)

**Supplementary Table 6e.** Comparative analysis of endoscopic postoperative management and outcomes in patients who underwent a re-resection between the French (n=146) and American cohort (n=222)

| Variables | French cohort (n=146) | American cohort (n=86) | p-value |
| --- | --- | --- | --- |
| One year follow-up available^**^,  *n(%)*  Endoscopic evaluation performed (<1 year postoperatively)    *Median time to first endoscopy (in months), median (IQR)*  Available modified Rutgeerts’ score at first postoperative ileocolonoscopy, *n(%)*  *i0*  *i1*  *i2a*  *i2b*  *i3*  *i4*  Endoscopic postoperative recurrence (≥i2b), *n(%)*  Severe endoscopic postoperative recurrence (≥i3), *n(%)* | 138 (94.5)  108 (78.3)  6.5 (6.1 – 7.7)  107 (99.1)  29 (27.1)  17 (15.9)  12 (11.2)  25 (23.4)  11 (10.3)  13 (12.1)  49 (45.8)  24 (22.4) | 75 (87.2)  44 (58.7)  6.7 (6.2 – 9.9)  42 (95.5)  21 (50.0)  7 (16.7)  2 (4.8)  5 (11.9)  4 (9.5)  3 (7.1)  12 (28.6)  7 (16.7) | **-**  0.003  **-**  **-**  **-**  **-**  **-**  **-**  **-**  **-**  NS  NS |

**Abbreviations**. NS = not significant (p-value >0.05)

**Supplementary Table 6f.** Comparative analysis of endoscopic postoperative management and outcomes in patients who underwent a re-resection between Canadian cohort (n=103) and the American cohort (n=222)

| Variables | Canadian cohort (n=45) | American cohort (n=86) | p-value |
| --- | --- | --- | --- |
| One year follow-up available^**^,  *n(%)*  Endoscopic evaluation performed (<1 year postoperatively)    *Median time to first endoscopy (in months), median (IQR)*  Available modified Rutgeerts’ score at first postoperative ileocolonoscopy, *n(%)*  *i0*  *i1*  *i2a*  *i2b*  *i3*  *i4*  Endoscopic postoperative recurrence (≥i2b), *n(%)*  Severe endoscopic postoperative recurrence (≥i3), *n(%)* | 44 (97.8)  39 (88.6)  6.2 (5.2 – 8.5)  38 (97.4)  14 (36.8)  10 (26.3)  4 (10.5)  9 (23.7)  1 (2.6)  0  10 (26.3)  1 (2.6) | 75 (87.2)  44 (58.7)  6.7 (6.2 – 9.9)  42 (95.5)  21 (50.0)  7 (16.7)  2 (4.8)  5 (11.9)  4 (9.5)  3 (7.1)  12 (28.6)  7 (16.7) | **-**  <0.001  **-**  **-**  **-**  **-**  **-**  **-**  **-**  **-**  NS  0.037 |

**Abbreviations**. NS = not significant (p-value >0.05)

**Supplementary Table 7.** Multivariable logistic regression models including country in relation to risk of endoscopic postoperative recurrence (modified Rutgeerts’ score ≥i2b) and severe endoscopic postoperative recurrence (modified Rutgeerts’ score ≥i3) with France and Canada as a reference^*^

| **Endoscopic recurrence** | | | |
| --- | --- | --- | --- |
| ***Country*** | **aOR** | **95% CI** | **p-value** |
| France  *Netherlands*  ***Canada***  ***United States of America*** | REF  0.92  **0.56**  **0.53** | –  0.68 – 1.24  **0.35 – 0.91**  **0.33 – 0.83** | –  0.590  **0.019**  **0.006** |
| Canada  *Netherlands*  ***France***  *United States of America* | REF  1.64  **1.78**  0.94 | –  0.99 – 2.72  **1.10 – 2.88**  0.51 – 1.72 | –  0.055  **0.019**  0.838 |
| **Severe endoscopic recurrence** | | | |
| ***Country*** | **aOR** | **95% CI** | **p-value** |
| France  ***Netherlands***  *Canada*  *United States of America* | REF  **0.53**  0.54  0.74 | –  **0.34 – 0.81**  0.27 – 1.11  0.40 – 1.34 | –  **0.003**  0.096  0.318 |
| Canada  *Netherlands*  *France*  *United States of America* | REF  0.97  1.84  1.36 | –  0.45 – 2.10  0.90 – 3.78  0.57 – 3.23 | –  0.933  0.096  0.492 |

***** models adjusted for sex, age and disease duration at surgery, smoking status at surgery, prior small bowel resection, lines of preoperative biologicals, disease location and severity (Montreal L/B/p+), postoperative prophylactic medication and time to ileocolonoscopy

**Supplementary Figures**

**Supplementary Figure 1.** Distribution of endoscopic outcomes in relation to the high-risk features according to the European guidelines (9)

**Supplementary Figure 2.**  Distribution of endoscopic outcomes in relation to the number of high-risk features according to the European guidelines per country (9)
